# A primary care pharmacogenetic precision medicine pilot based on specific Māori tribal ethical frameworks and principles

**DOI:** 10.1007/s12687-026-00914-7

**Published:** 2026-06-28

**Authors:** Stephen P. Robertson, Benjamin J. Halliday, Rhonda Tibble, Caroline Koia, Tuta Haereroa, Elizabeth Goodin, Ben Curran, Claire E. Rye, Helen Wihongi, Ben Te Aika, Joep de Ligt, Donia Macartney-Coxson, Nick Jones, Jun Huh, E. Owen Perkins, Matt Pestle, Kenny Zhao, Martin A. Kennedy, Patrick A. Gladding, David M. Markie, Cristin G. Print, Phillip L. Wilcox, Huti Puketapu-Watson

**Affiliations:** 1 Department of Paediatrics and Child Health, University of Otago, Dunedin, Aotearoa New Zealand; 2 Ngāti Porou Oranga, Kaiti, Gisborne, Aotearoa New Zealand; 3 Department of Molecular Medicine and Pathology, University of Auckland, Auckland, Aotearoa New Zealand; 4 Computational Biology Group, Children’s Cancer Institute of Australia, Sydney, Australia; 5 Digital Services, University of Auckland, Auckland, Aotearoa New Zealand; 6 Health NZ Research/Rangahau Hauora Aotearoa, Te Whatu Ora Aotearoa, Wellington, Aotearoa, New Zealand; 7 Te Ira Tātai Whakaheke Trust, Aotearoa, New Zealand; 8 Research and Enterprise Office, University of Otago, Dunedin, Aotearoa New Zealand; 9 New Zealand Institute of Public Health and Forensic Science, Porirua, Wellington, Aotearoa New Zealand; 10 Department of Pathology and Molecular Medicine, University of Otago, Christchurch, Aotearoa New Zealand; 11 Department of Cardiology, Te Whatu Ora, Auckland, Aotearoa, New Zealand; 12 Department of Pathology and Molecular Medicine, Faculty of Medicine Dunedin, University of Otago, Dunedin, Aotearoa New Zealand; 13 Maurice Wilkins Centre for Molecular Biodiscovery Auckland, Auckland, Aotearoa New Zealand; 14 Department of Mathematics and Statistics, University of Otago, Dunedin, Aotearoa New Zealand

**Keywords:** Māori ethical frameworks, Precision medicine, Pharmacogenetics, Drug response, Ancestry, *CYP2C19*

## Abstract

Indigenous tribal communities are frequently underserved by genomic medicine because they are under-represented in research, often live in remote locations and have ethical and cultural perspectives that vary from westernised standpoints. The Rakeiora platform, a permissioning and analytical environment that incorporates Māori perspectives and ethical frameworks on how precision medicine research is best conducted was used to host a pharmacogenetic exemplar project to demonstrate proof of principle in a tribal community. The project was prospectively co-designed alongside community representatives through an extensive series of kanohi ki te kanohi (face-to-face) consultation hui, drawing on the strengths, insights, and aspirations of the community. Narratives socialising the study were metaphorically located in tribal-specific histories to improve participation. The cohort of 148 Māori individuals consented to have variants in *CYP2C19* extracted from whole genome sequence datasets and correlated to the medicines they were prescribed and had dispensed over a 12-month period. Genotyping indicated that 7% of the cohort were genotypically predicted to be “poor metabolisers” and 11% “rapid metabolisers” of at least one drug known to be processed by CYP2C19. Based on international guidelines, 40% of prescriptions could have benefited from knowledge of the participant’s *CYP2C19* genotype through either dose adjustment or prescription of an alternative drug. The study population was broadly supportive of research into precision medicine, but the majority wished to retain ongoing dynamic consent practices mediated by trusted local figures.

**Supplementary Information:**

The online version contains supplementary material available at https://doi.org/10.1007/s12687-026-00914-7.

## Introduction

The central principle of precision medicine is that data from multiple sources relevant to an individual should be easily accessible to guide healthcare decisions at the point of care. Although it could be asserted that healthcare in various cultural contexts has always, for the most part, been personalised and strives to be precise, a key difference in this new era is the employment of data informatics and analysis at scale (Evans et al. 2024). Research into operationalising this is accelerating and is employing datasets drawn from electronic clinical records, administrative information and genomic datasets. Given this rapid scale-up in the size and amount of data sourced on individuals and analysed using algorithms in precision medicine research, ethical concerns focusing on data security, discoverability, and privacy continue to grow (Aitken et al. 2016).

Added to these generic ethical concerns are the voices of indigenous communities (D’Angelo et al. 2020; Garrison et al. 2019; Arbour and Cook 2006; Robertson et al 2018). Indigenous peoples recognise, and have described in detail, ethical perspectives and frameworks that demand that their data and information are handled differently than the way prescribed by Westernised standpoints (Beaton et al. 2017; Carroll et al. 2022). These principles include (a) insistence that the contributing communities in such research can independently identify a benefit to their people for the research; (b) that the research actively involves not only their participation but also direct control over its conduct; (c) that researchers have an ongoing and direct line of communication to participating communities so that there is reciprocity in communication and lines of responsibility are explicitly laid out, and finally, (d) that the unique ethical precepts surrounding protocols of a cultural nature such as sample and data handling and dissemination of results are an intrinsic part of ethical review of the healthcare implementation or research proposition (Carroll et al. 2021). Consequently, to meaningfully implement these principles, prospective co-design of studies is mandatory with genuine involvement of indigenous people from the outset as integral members of research teams (Robertson et al. 2018).

Māori, the indigenous people of Aotearoa New Zealand, have been globally prominent over the last 15 years in enunciating clear ethical principles that should underpin genomics research within their communities (Hudson et al. 2016a, b, 2020; Robertson et al. 2018; Rye et al. 2025). Fundamental precepts include the prescription of tikanga (protocols) around the handling of samples, the primacy of reciprocity in anticipating and demonstrating benefits from the research and the importance of recognising that the continuum of responsibility that exists between participants, their samples and data is not extinguished by anonymisation or merging of individuals datasets into aggregated cohorts. To implement these principles and to create an analytical computational environment supported by integrated protocols to enable precision medicine research in Aotearoa New Zealand, the Rakeiora project was launched in 2018. The shape and functionality of the data handling environment and the protocols surrounding it have been described elsewhere (Rye et al. 2025). The analytical environment consists of a virtual “walled garden” into which datasets can be imported from a diversity of sources by researchers who have been vetted and granted access to the system (i.e. given a passport). Researchers with passports can then apply to work on individually permissioned and approved projects, granted though a visa application protocol. Analysis occurs within the walled garden using containerised tools and no underlying data can be exported, only the results of the data analysis.

Since this project involved partnering with a tribally-based Indigenous health provider, a community-based participatory co-design approach (Udoewa 2022) was considered mandatory by tribal partners. Co-designs such as radical participatory design empower and prioritise communities rather than external actors such as researchers. In this context, co-design demands that the perspectives, culture, ethical frameworks and priorities of the community are central to the design of the research protocols. A key rationale is that tribal members are more likely to identify with study goals if they are articulated in a manner that is relatable to prospective tribal participants and open to modification with their involvement. The benefits that accrue by adopting this approach are the incorporation of mātauranga-a-hapori (traditional knowledge held by that community) into the explanations and framing of the research proposition, a clearer definition of the aims of the work that align with community priorities and the institution of local arrangements that formalise lines of accountability and communication.

Here we describe the first exemplar project using real-world data on individuals based within a primary care healthcare environment, to pilot the Rakeiora system. In this study, individuals were recruited using a co-design approach based on a community based participatory design (Wilcox et al. 2008). It describes a pharmacogenetic study of 148 people involving the co-import of gene sequence subsets from remotely stored whole genome sequence (WGS) data, prescription information from the electronic health record (EHR) of these same individuals, and matching drug dispensing data held by Government agencies on these participants. The aim was to calculate the rates of prescription (and dispensing) of drugs predicted to be differentially metabolised according to previously identified diplotypes in a well-studied pharmacogene, *CYP2C19.* This pilot project demonstrates the tractability of employing a precision medicine platform to conduct research that aligns with, and is guided by, Māori ethical frameworks. This work also indicates that prescribing practices could be measurably improved if pharmacogenetic data were available at the point of care.

## Methods

Co-design of this project was undertaken with a group of experts in mātauranga and/or tikanga Māori (Māori knowledge and ethics) and began with processes drawing largely upon various tikanga-based frameworks developed for various gene technology applications. For the purposes of this pilot project, the staff of the Research Unit within Ngāti Porou Oranga (NPO), Te Rangawairua o Paratene Ngata Research Centre, were assigned the status of local liaison and kaitiaki (custodians and overseers of a treasured resource).

### Ethical approval

Ethical approval for the project was obtained from the New Zealand Health and Disability Ethics Committee 20/STH/185 and the Ngāti Porou Hauora (now Ngāti Porou Oranga; NPO) Ethics Committee.

### Co-design of community engagement and recruitment

An intrinsic element to the design of this project was the employment of participatory action co-design principles (Udoewa 2022) to shape methodology, protocols, and communication so that it reflects tribal mātauranga (knowledge originating from Māori ancestors, including the Māori world view, perspectives and practices including that which is specific to the tribal group itself) and tikanga (ethics). This began with the assembly of a team that included community health leaders and cultural experts, particularly those with deep knowledge of Ngāti Porou language, tikanga and mātauranga (RT, HW, TH). A strong element of the formulation of the project - its aims, protocols and rationale - was the contextualisation of the project to Ngāti Porou lore through the deployment of tribal tikanga. An example of this leading to a relatable scientific proposition was the naming of the analytical system itself - Rakeiora - a metaphorical framing of a scientific navigation through previously uncharted waters (Supplementary file). The imagery inherent in this framing incorporated ocean-going craft (waka hourua), ancestral voyagers and contributions by male and females alike - all related in local Māori dialect (te reo Ngāti Porou). The resulting booklet (Supplementary file) was distributed to attendees at the community hui.

A series of three community hui (community meetings to discuss issues of mutual relevance) were held across the Tairāwhiti district to communicate the intent of the project and invite input into the design of the project. The hui were facilitated by a local cultural expert who used a combination of visual aids, role play and small group discussions to assemble attendee priorities, perceptions and ideas. This included scoping potential benefits and risks for the research, reviewing and designing the content of the consent form and information documents that would be presented to participants, along with the questionnaire that would also be presented to them and recommendations about how study results could be converted into health records for ongoing use.

A local recruitment team, consisting of members of the Ngāti Porou community, were employed to approach individuals over the age of 18 years and who were enrolled with NPO for delivery of their healthcare to participate in the project through meetings in their homes or at community functions. The recruiter needed to iteratively develop and test approaches for engaging with prospective study participants, on a kanohi-ki-te-kanohi (face-to-face) basis.

Written informed consent was obtained including permission to access their NPO EHR. Additionally, prospective participants were informed that if they agreed to participate, administrative datasets held by Government organisations would be accessed to obtain a record of medicines prescribed to them over the calendar year 2021. All data and samples were de-identified through the application of a numeric identifier at enrolment. Prescription and administrative data were accessed through secondary linkage to their National Health Identifier (NHI), a nationwide universal identifier for the delivery of healthcare. A request was made to each participant to nominate a family or community member to make decisions in the future around their participation in the research and handling of their data should they become unable to act for themselves. Specific consent was obtained for only the current pharmacogenetic study, but a questionnaire was administered that explored perspectives on future more open-ended participation in other precision medicine genomics research.

### Sample acquisition and handling

A saliva sample was collected directly into preservative (Oragene, DNAgenotek, Ottawa, Canada) with each participant offered the opportunity to recite a karakia (an incantation to culturally contextualise the spiritual nature of the activity) before dispatch of the sample. Once samples were processed at a central laboratory further karakia were recited over the final samples (and byproducts produced during their preparation) before dispatch for sequencing at the Garvan Institute, (Darlinghurst, Australia). A Memorandum of Understanding was established with the Garvan Institute to ensure that all unused DNA and all data generated from sequencing would be returned to Aotearoa New Zealand with no data left on host servers at the completion of the study.

### Whole genome sequencing

Sequence data was returned as paired-end FASTQ files, with a read length of 150 bp. Alignment of sequence reads to GRCh38 was performed using Burrows-Wheeler Aligner (v0.7.17) applying the MEM algorithm with output in BAM format. DeepVariant (v1.5.0) was used for variant calling to produce single sample VCF files. VCF and BAM files were stored securely in servers hosted by the University of Otago.

### Platform characteristics and design

The architecture of the Rakeiora permissioning and analytical platform have been described in detail elsewhere (Rye et al. 2025). Briefly, it consists of an analytical environment termed a “walled-garden”, into which permissioned researchers can input datasets to analyse under pre-agreed protocols. A system of passport-granting registers a researcher as a user of the system and records their current data access permissions. A genomic data retrieval tool, htsget, facilitates import of genomic data from remote locations and a bespoke application (Resource Entitlement Management System – REMS – CSC, Helsinki, Finland) manages consent, approval, and access rights to bioinformatic resources. Records from administrative datasets, electronic health records, and whakapapa (a Māori term for genealogy including genetic and cultural endowments transmitted from previous generations) information can be uploaded in SQL format. In this case using participants’ NHI, data was ingested into a participant data management platform (ALEA) and converted to study identifiers within a privacy portal. Representatives of NPO were mandated by participants at consenting to act as the kaitiaki of the genomic, medical, and administrative datasets used in this study and for granting permission for ingress of these data into the analytical environment for analysis.

### Extraction and ingestion of genomic, prescription and administrative data into the walled-garden analytical environment

Data summarising genetic variation across the genomic regions encompassing the gene body of *CYP2C19* (in BED format), including 2 kb of flanking sequence, was extracted from single sample VCF files, exported into the walled-garden using htsget, and merged for downstream analyses. Data for drug prescribing performed through NPO for a single calendar year (2021) were extracted from participants’ EHR and uploaded into a secure SQL database where they were assigned study codes. Similarly, an application for access to an administrative dataset of all dispensed medicines held by the New Zealand Government through PHARMS warehouse (a government held repository of all dispensed medicines, indexed by NHI; https://www.health.govt.nz/nz-health-statistics/national-collections-and-surveys/collections/pharmaceutical-collection*)* for the 148 participants over the calendar year 2021 produced a matched record of dispensed medicines for each participant, coded identically to the EHR prescription data and uploaded and de-identified pending analysis.

### Joint analysis of the datasets within the Rakeiora analytical environment

**Fig. 1:**
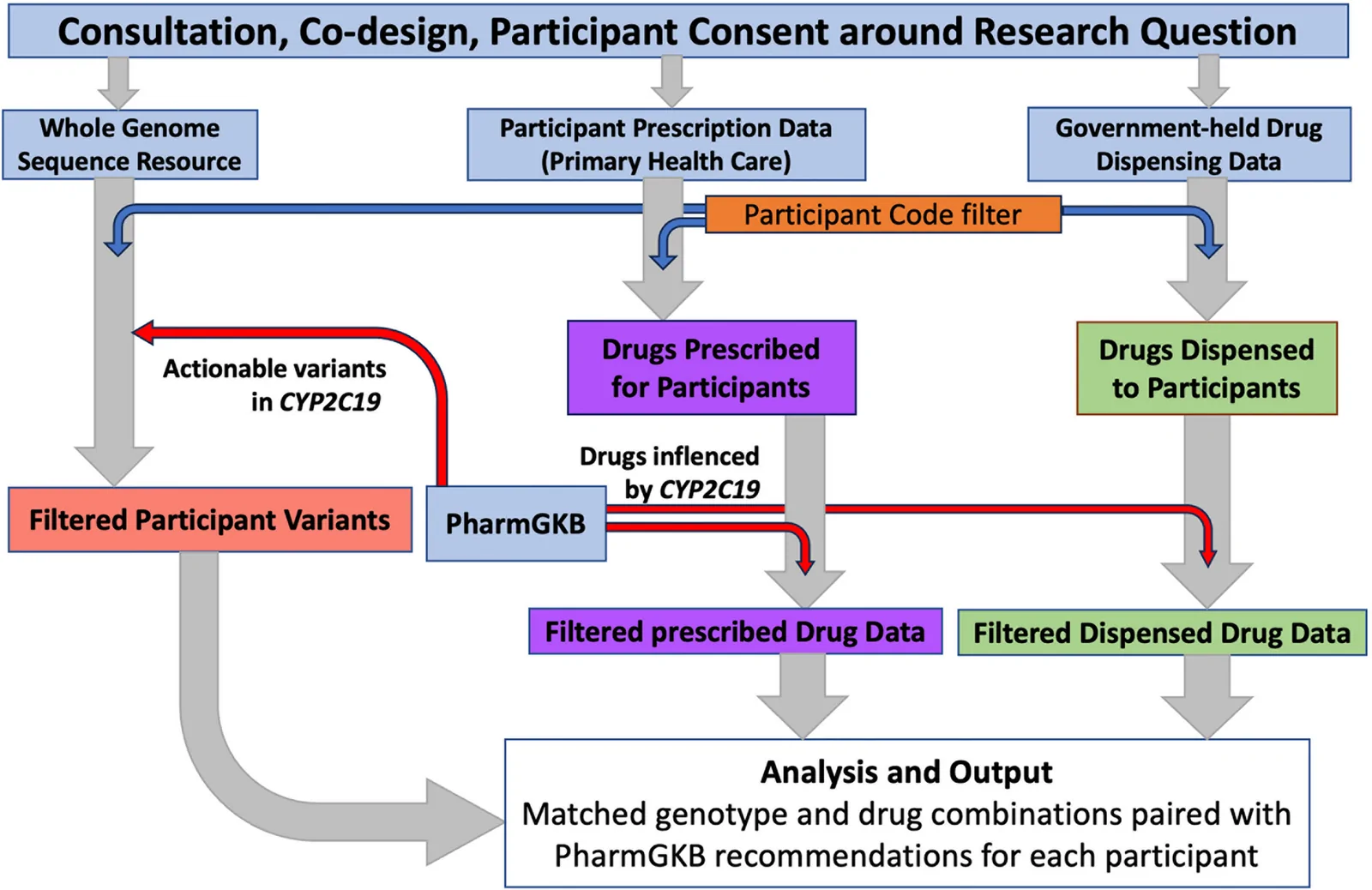
Workflow diagram for the pharmacogenetic precision medicine exemplar. Three sources of data (whole genome sequences, prescription data and drug dispensing data) were filtered and then integrated within the Rakeiora analytical environment to calculate the incidence of drug exposure/genotype combinations. The PharmGKB database can be found at https://www.clinpgx.org/

A snakemake workflow (https://github.com/BenjHalliday/rakeiora-public-pharmacogenomic) was constructed to co-analyse the genomic, prescription and dispensing data within the Rakeiora analytical environment (Fig. 1). Three common *CYP2C19* alleles have been shown to reliably demonstrate clinically meaningful influences on the metabolism of medicines often prescribed in primary care - CYP2C19*2/*3/*17. The reference allele is denoted as CYP2C19*1. Alleles CYP2C19*2 and CYP2C19*3 (NG_008384.3: g.[17687 A > G;24179G > A;85186 A > G] and NG_008384.3: g.[22973G > A;85186 A > G] respectively) are both loss-of-function alleles meaning that they confer functional insufficiency to the enzyme and are characterised null alleles. The CYP2C19*17 allele (NG_008384.3: g.[4220 C > T;85186 A > G]) refers to an over-expression “gain-of-function” allele and contributes to a rapid-metaboliser phenotype because of enhanced transcription at the *CYP2C19* locus.

The accuracy and analytical validity of *CYP2C19* diplotypes, was confirmed using a clinically accredited provider (Theranostics, Grafton Clinical Genomics, Auckland, New Zealand) using an Agena MassARRAY. Clinically relevant genotype-drug exposures were sourced from https://www.clinpgx.org/gene/PA124 (Lee et al 2022) Participants were notified of their results by letter and clinically validated results were returned to the Ngāti Porou Oranga primary healthcare practitioner by letter for incorporation into the participant’s EHR.

### Participant questionnaire design

To assess the acceptability and generalisability of this precision medicine methodology a questionnaire was developed and presented to participants regarding their views on the governance of data generated in studies such as this for precision medicine research. Perspectives that were heterogeneous across communities, issues that directly impacted on community arrangements for managing cultural risks (e.g. storage of data, responsibility for data use and security after death of a participant) and exploration of factors that could be improved upon in moving more expansively to fully operative precision medical practice were explored. Draft questions were then modified and finalised by the co-design team. The finalised questionnaire (Supplementary file) was given to study participants by the recruiter after consent to participate was provided.

## Results

Three hui were held at separate locations within the tribal region, Tikitiki, Ruatoria and Uawa. Key elements of the design of the protocol which were central to the success of fully informed and consented participation in the project included.

- A full exploration of the necessity for the arrangements put in place to mitigate cultural risk for samples going offshore including who was responsible for the kaitiaki of the DNA samples and the data derived from them.
- Establishment of trust and comfort with the research proposition was improved with recognition that the recruiter was a member of the local community who could recognise the importance of whakapapa and had sufficient time to be flexible with time and location with recruits.

A cohort of 148 individuals (100 female and 48 male; age range 21–90 years, median 60 years) was assembled for this pilot project. All identified as Māori and all were enrolled for their healthcare with Ngāti Porou Oranga. All participants consented to submitting a saliva sample and 147 filled out all or some of the questionnaire. Once the prescription and dispensing data were extracted from the NPO EHR and Pharms Warehouse repositories and ingested into the privacy portal, an application from the analytical team to the kaitiaki of these datasets (the leadership of Te Rangawairua o Paratene Ngata Research Centre) was made for the transfer of it, along with the nominated relevant genomic data, into the analytical walled-garden for analysis.

### *CYP2C19* genotype frequencies

**Fig. 2:**
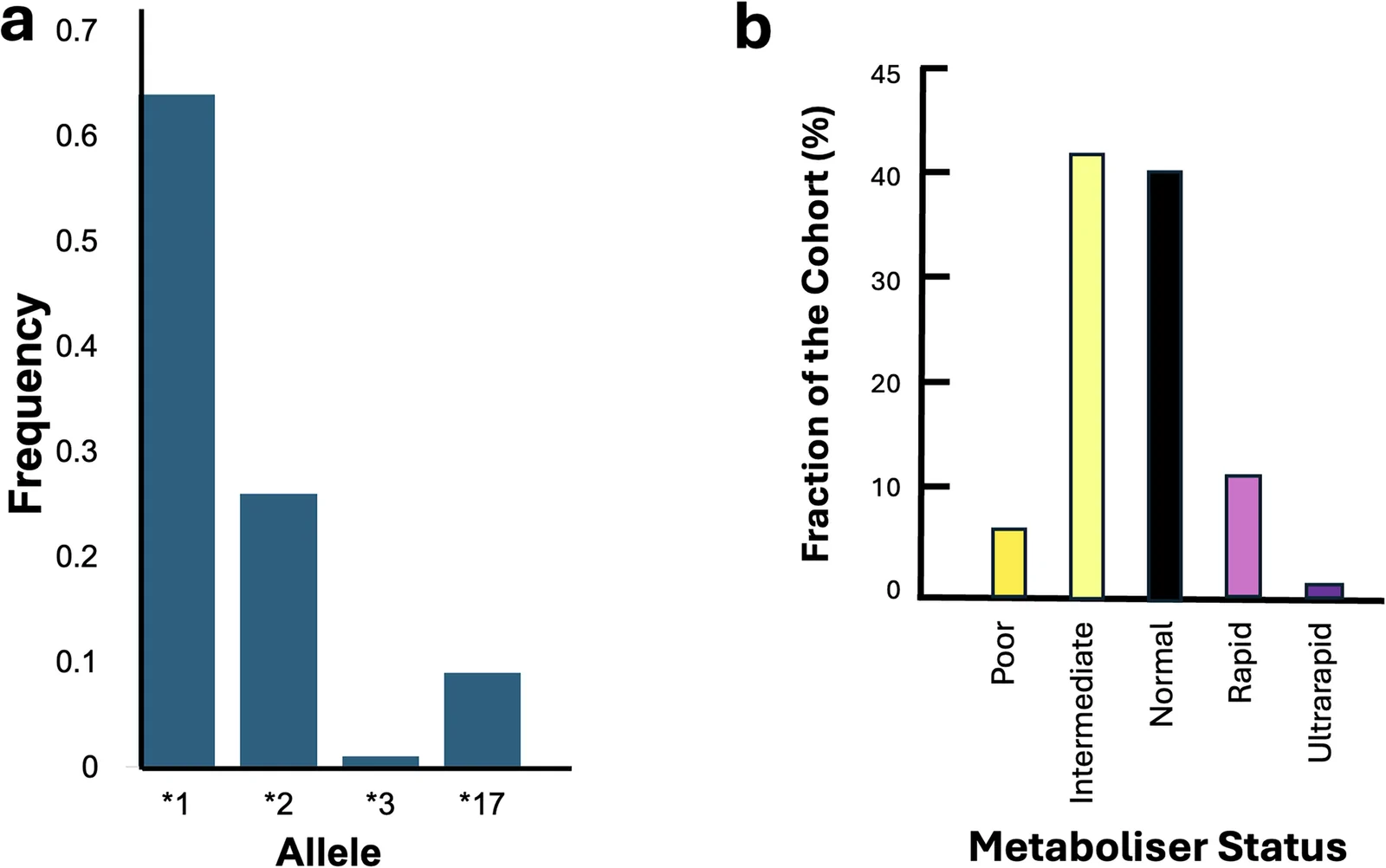
*CYP2C19* allele frequencies and metaboliser status across the cohort (n = 148). (a) Frequency distribution for *1, *2, *3 and *17 alleles; (b) Percentage of the cohort with diplotypes that predict poor (*2/*2), intermediate (*1/*2, *1/*3, *2/*17), normal (*1/*1), rapid (*1/*17) and ultra-rapid (*17/*17) metaboliser status

Three non-reference *CYP2C19* alleles were genotyped by the workflow. All 148 diplotypes that were called from the genomic data via the workflow and independently assayed on separate DNA samples were concordant. Genotypic data from all 148 participants is presented in Table 1; Fig. 2b. Individuals with a genotype of *1/*1 (*n* = 60; 40%) have unmodified CYP2C19 activity, while those with diplotypes *2/*2, *3/*3, and *2/*3 were assigned as poor metabolisers (*n* = 10; 7%), those with *1/*2, *1/*3, *2/*17 or *3/*17 diplotypes as intermediate metabolisers (*n* = 61; 41%), those with a *1/*17 genotype as rapid metabolisers (*n* = 16; 11%) and the individual with a *17/*17 genotype having an ultra-rapid metaboliser phenotype (*n* = 1; 1%).

**Table 1:** Allele and Genotype frequencies across the cohort (*n* = 148)

Diplotypes			Alleles			
	Number	Proportion (%)	*1	*2	*3	*17
*1/*1	60	40	120	0	0	0
*1/*2	51	35	51	51	0	0
*1/*3	2	1	2	0	2	0
*2/*2	10	7	0	20	0	0
*1/*17	16	11	16	0	0	16
*2/*17	8	5	0	8	0	8
*17/*17	1	1	0	0	0	2
Total	148	100	189	79	2	26
Allele frequency			0.64	0.26	0.01	0.09

**Fig. 3:**
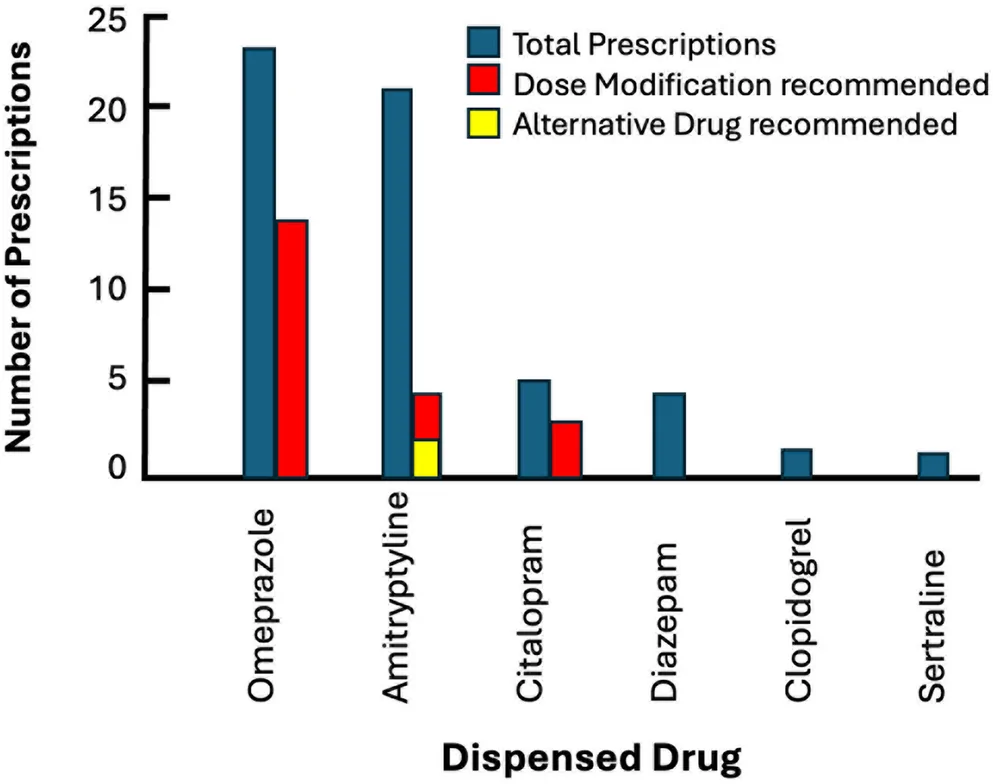
Dispensed drugs over a 12-month period and prescription recommendations following genotyping. Six drugs were prescribed to participants that are metabolised by CYP2C19 (blue). Potential alterations to prescriptions after consideration of individual genotypic status included alteration in initial or maintenance dosages (red) or prescription of an alternative agent (yellow)

Certain *CYP2C19* diplotypes predict either non-responsiveness to certain commonly prescribed medicines (e.g. clopidogrel requires active CYP2C19 to convert the drug to its active form, hence poor metabolisers are predicted to respond suboptimally to this drug) or alternatively imply clinical benefits from an alteration in prescribed dose (e.g. poor metabolisers prescribed the proton pump inhibitor omeprazole may benefit from dose reduction). A dynamic database of recommended clinical guidelines https://www.clinpgx.org/gene/PA124 was consulted for these specific recommendations. In this study, prescriptions for the following drugs were identified – citalopram, amitriptyline, omeprazole, clopidogrel, diazepam, and sertraline. No participant was prescribed clozapine, escitalopram, or esomeprazole over the period of study. Summative data were generated that listed the prevalence of unfavourable or clinically actionable combinations of dispensed medicines and diplotypes (Fig. 3).

All prescribed medications were registered as dispensed in the Pharms Warehouse database. Four instances of medicines dispensed to participants did not have a matching prescription issued by an NPO practitioner but were included in the analysis.

The most common alternate allele noted was CYP2C19*2 (cohort allele frequency (AF) = 0.26). Consequently, many clinically actionable interactions were identified in those whose diplotypes assigned them as intermediate or poor metabolisers (Fig. 3) and therefore potentially at risk of toxicity (or non-responsiveness in the case of clopidogrel) if prescribed some drugs at conventional doses. Of all the drug prescriptions recorded here (*n* = 53), 21 of these (40%) were for participants with a *CYP2C19* diplotype for which modification of the conventional dose or, in two instances, a substitution of one drug for another, could be considered.

Individuals with the genotype *1/*17 (*n* = 16) or *17/*17 (*n* = 1; Fig. 2) face a different situation with most of the drugs considered in this study – enhanced metabolism and a shorter half-life of the ingested drug - a situation that also could require adjustment in prescribing practice. Because of the low frequency of CYP2C19*3 alleles, no disadvantageous genotype-drug prescription combinations relating to this allele were observed in this study. The most problematic potential genotype-drug combination - that of a poor metaboliser who is prescribed clopidogrel (a drug that requires activity of this enzyme to convert this drug to its active form) - was not observed in this study.

### Questionnaire results

An essential element of co-design of similar research projects that propose to use the Rakeiora system in the future is understanding the preferences that Ngāti Porou communities have on data handling and consent practices. A questionnaire was administered at enrolment that combined direct questions in addition to the opportunity to offer perspectives using free text. Answers revealed significant heterogeneity in perspective, comfort and priority-setting for future precision medicine research in the primary care setting. A majority (79%) said they would consent for other projects for future precision healthcare but most (73%) would wish to personally consent for future projects with fewer (39%) comfortable for a nationally mandated body to make this decision on their behalf. A majority (62%) voiced comfort with their data continuing to be used after their death but overwhelmingly only if a whānau (family) members were personally involved in decision-making. A minority (34%) were agreeable to the sharing of their data with overseas researchers but only if there was direct involvement of Aotearoa New Zealand researchers with accountability to their community.

There was substantial comfort in having NPO fill the role as kaitiaki and priority-setting agency but less comfort in having bodies more removed from NPO (such as mandated national committees) fulfilling this function. The centrality of addressing and observing local tikanga in formulating study design was a strong message as was the importance of acknowledging and observing the various embodiments of whakapapa that include pedigree information, samples, and genomic data.

## Discussion

Engagement of Māori communities from the outset in the co-design and delivery of research and the alignment of research practice with local tikanga results in stronger participation, more relevant outcomes and a stronger sense of ownership in these kaupapa (project) (Robertson et al. 2018). The design of the Rakeiora precision medicine research platform sought to embed mātauranga Māori in the design and delivery of research into this fast-evolving area of healthcare. Features of this research which set it apart and distinct from conventional research practices include the employment of cultural protocols around the collection, storage and use of samples and data, the use of community-based consent practices, project leadership by local trusted figures, specific adherence to data sovereignty through locally developed protocols and the appointment of trusted community figures to function as kaitiaki over the research data and samples. In matters relating to the kaitiakitanga (the practice and principles surrounding custodianship) that must surround the use of samples and data, especially after the death of the participant, a questionnaire explicitly sought to foster an ongoing conversation on such matters that have yet to be explored deeply in the context of precision medicine research with longitudinal designs. In this respect an understanding and agreement of how certain fundamental aspects of the precision medicine research proposition should be safely deployed are still evolving.

In co-designing the primary care research plan, Ngāti Porou communities spoke strongly to the necessity of producing results that were of direct and immediate benefit. This imperative lies in addition to the strong support they voiced in exploring the establishment of a precision medicine research platform. Since the analysis that occurred within the Rakeiora environment is de-identified and no raw or personalised data can leave this system, a separate analysis was performed with the sole aim of returning benefit to the participants.

Choosing to study the relationship between well-characterised variants in *CYP2C19* and drug exposures was driven by the clinical validity of the practice in both in Aotearoa New Zealand (Gladding et al. 2010; Larsen et al. 2015; Panattoni et al. 2012) and offshore (El Rouby et al. 2020; Zabalza et al. 2012; Bousman et al. 2023). *CYP2C19* allele frequencies for alleles vary significantly across Oceania, with high frequencies observed in individuals with Melanesian ancestry and lower rates across Polynesia (Helsby 2016). Previous statements that Oceanian populations are at risk of medication-related harms related to the high prevalence of *CYP2C19* null alleles have often failed to consider the broad variability in allele frequencies across the Pacific, with Polynesian people being falsely assumed to have similar allele frequencies to people with Melanesian ancestry (Helsby 2016). These data are consistent with previous observations that the *2 allele is present at a higher rate in Māori than European New Zealanders (Lea et al. 2008; Gladding et al. 2010) and that the allele frequency of the gain-of-function *17 allele is correspondingly lower. Around 7% of participants in this study are poor metabolizers, a greater than two-fold over-representation compared to populations with European ancestry (Petrovic et al. 2020). The data presented here underscores the importance of deepening our understanding of the genetic diversity present within Māori (and Pacific) peoples to ensure that precision health is delivered on an equitable and accurate footing in Aotearoa New Zealand (Helsby 2016).

The potential consequence of inefficacy of clopidogrel due to failure to activate the prodrug via CYP2C19 action is thrombosis, which can be life-threatening (Martin et al. 2020). Some evidence exists to promote pharmacogenetic-guided prescription in this clinical scenario (Bousman et al. 2023). For some drugs with a high therapeutic index (for example, omeprazole and amitriptyline), *CYP2C19* genotype informed prescribing may not be so advantageous, but for others the effects could be clinically significant, for example the anti-depressant citalopram (Hicks et al. 2015; Bousman et al. 2023). Given this complexity, alongside the allele frequencies observed in this project, pharmacogenomic information could usefully inform future clinical practice in Aotearoa/New Zealand. Now that the capability has been developed to analyse pharmacogenes and cross-reference those data with prescribed and dispensed drug records using NHI records, approaches towards implementation of this practice should be developed to enhance prescribing, maximise therapeutic benefit and minimise harms using a similar precision medicine approach to that described here (Claw et al. 2024). Once a more robust understanding of allelic diversity is obtained across a broader range of pharmacogenes (e.g. *CYP2D6*) full genome sequences may not be required to perform research like this, and studies could be powered to very high levels using affordable research designs. Although this study did not seek to address other factors that could influence drug response in Māori such as the existence of novel variation in *CYP2C19* (Hitchman et al. 2026) or the influence of ancestry-specific epistasis, it does provide a methodological template to more systematically address these issues in future larger studies. By using a community-based participatory design framework this study has generated clinically relevant information. Whether this magnitude of effect can be implemented into clinical practice remains to be seen but the use of participatory design from the outset should embed levels of comfort and acceptance within communities that will enhance the chances of uptake to similar levels. Others have strongly made the case for the importance of studying pharmacogenetic parameters in indigenous populations in the context of community centered methodologies such as those adopted here (Claw et al. 2024).

## Supplementary Information

Below is the link to the electronic supplementary material.


Supplementary Material 1: File 1. The Rakeiora Study booklet



Supplementary Material 2: File 2. Questionnaire for Participants of the Study


## Data Availability

The survey data that support the findings of this study are available from the authors after reasonable request. For DNA sequence data relating to *CYP2C19* genotypes restrictions apply to the availability of these data, which were used under the ethical protocols established for the current study and so are not publicly available. The data are, however, are available upon reasonable request that aligns with conditions outlined with the ethical approval and consent conditions granted for this study.
