# Supplementary material for: A primary care pharmacogenetic precision medicine pilot based on specific Māori tribal ethical frameworks and principles
Source: J Community Genet. 2026 Jun 28;17(4):79. doi: 10.1007/s12687-026-00914-7 (PMC13310208; doi:10.1007/s12687-026-00914-7)
Supplement: Supplementary file 1 — Supplementary Material 1: File 1. The Rakeiora Study booklet [file 12687_2026_914_MOESM1_ESM.pdf]

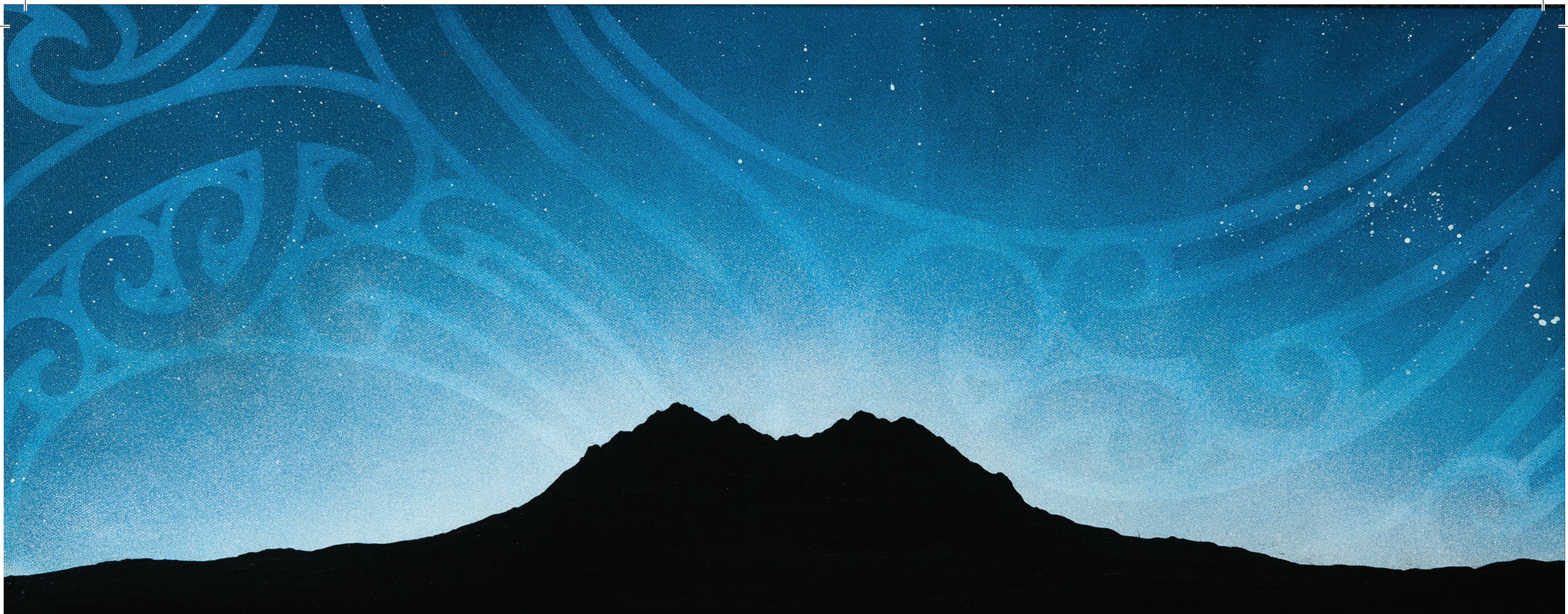

NGĀTI POROU HAUORA

The Rakeiora Project

"Whakakahāngia toū oranga
Hei whakaora tou nohanga I te ao nei"

"Strengthen your health, so your life in this world may be prolonged"

Kia hiwa ra Kia hiwa ra	*Be aware of where our health is*
Kia hiwa ra ki tenei tuku	*Be receptive to good health*
Kia hiwa ra ki tera tuku	*Be approachable to making modification*
Kia tu Kia oho Kia mataara	*Stand up! Be awake! Be watchful!*

E Hika ma Ngāti Porou whanui tonu tēna tatou.

E whai āke nei ko nga kohinga kōrero a te hunga matakite e rangahau ana te hauora a to tatou iwi.

Ko te hamara a te rōpu Rakaiora kei te kawe i tenei kaupapa.

Ko wa ratou kitenga hei whakamataara ake ia tatou kia āta tirotiro kei tewhea taumata ra te oranga a to tatou iwi i te ao nei.

The whakapapa of Rakeiora

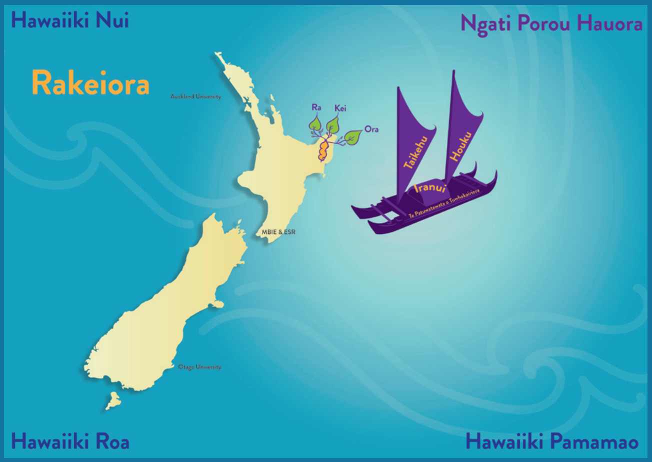

CREDIT: Melanie Tahata

Rakeiora is a descendant of Toi. He was a renowned Tohunga and navigator, through his leadership he was able to display his uncanny skilled knowledge of his surroundings, as a Tipuna Rakeiora depicts his wellness through his journey as a pathfinder. A visionary, he led his people to new horizons to discover new lands, places that would sustain life where they could stand and flourish. The Waka Hourua carried Rakeiora to the fulfilment of his visions and aspirations.

Genomics is the new horizon and within lies new grounds waiting to be explored yielding treasures that will enrich our lives. Ngāti Porou, along with our partners from University of Otago, University of Auckland, ESR, Genomics Aotearoa and MBIE want to chart a sure course in a safe way that protects our treasures for the benefit of all. **For more detailed information, refer to the koha written by Rhonda Tibble (page 12).**

All of us would like to think our doctor has the “full-picture” about us when making decisions about our healthcare. Not only about our symptoms and our test results but also our history - what medicines, treatments and lifestyle changes worked in the past, and which ones didn’t.

Part of this is knowledge about the health history of our whānau - because we all know that health conditions can run in families. Things would be a whole lot better for doctors if some of this “guesswork” in medicine was taken out.

Precision medicine aims to help improve the decision-making ability of doctors with systems that bring together your medical records, what you know about your whānau medical history, and your genetics. This type of medicine is new because it requires powerful computing resources and the ability to pull all the information together at the right time, in a safe way with all the support necessary for the doctor to make the right decision.

Aotearoa needs to develop this ability. To do that we need ways to draw relevant information together on a specific person, analyse it accurately and produce results that are meaningful, useful and precise. For us to get there it will take more research but to begin we need to establish the capability and test these systems under local conditions. That is what the Rakeiora Pathfinder Project aims to achieve.

Genomics and whakapapa

What are genes / what is genomics?

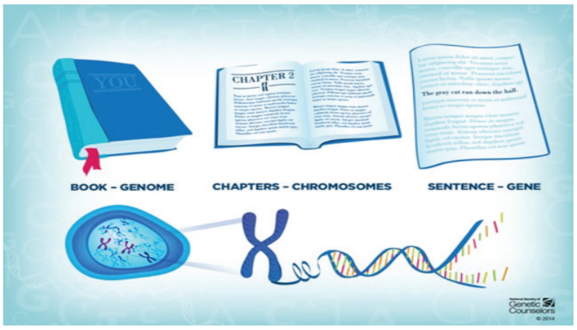

Genes are the instruction codes for our body. Think of genes as the many sentences that make up a book - one with lots of chapters. Much like whakapapa, these genes are inherited from our parents and our ancestors. Each gene carries a set of instructions for building individual characteristics, like eye and hair colour, and for conditions we can inherit.

Genes are made up of a substance called DNA. Everything we need to know is in the letters than make up that DNA – like the words which make up those sentences for the book.

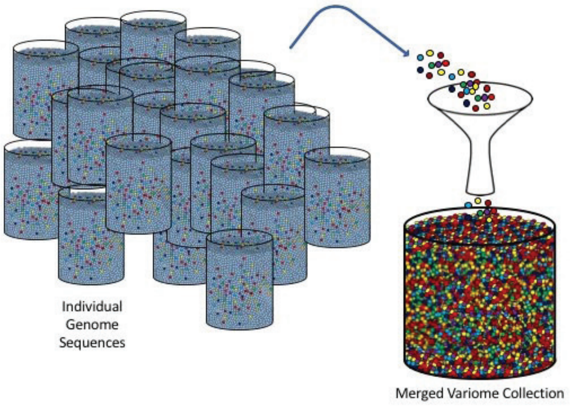

The full set of genetic instruction is called a genome – the entire book. Humans have around 20,000 genes in our genome. As with whakapapa, these genetic codes hold your family history – they are passed on to you through ancestors to your parents and on to your tamariki and mokopuna.

Inheritance means that parental genes are mixed in many different ways between family members. And that means relatives will have small but significant differences in the genetic codes. These few small differences, known as genetic variation, are enough to make each person unique.

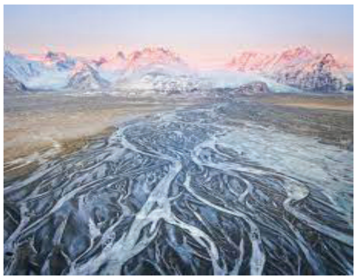

It's these unique combinations of variation in our genetic codes that points us in the direction of why some humans are more likely to have a particular disease than others.

DNA inherited from our tīpuna merges and divides into different combinations, just like a braided river that comes down from the mountain. Those combinations of whānau inheritance flow on in different forms onto our mokopuna.

Also passed on from our tīpuna are genes that improve or reduce our resilience to modern health conditions. Because everyone inherits a unique set of such genes, there is variation within and between whānau in resilience. This is also why we don't all look exactly the same, and also why our resilience to heritable condition varies. So to deliver personalised medicine (PM) for Māori, whakapapa is very important. Together with whakapapa, DNA will allow us to predict the resilience of nga uri whakatipu me nga uri ake nei: PM = whakapapa + DNA. Potentially, this information can inform whānau about conditions *before* they arise, so that interventions can be undertaken to improve resilience. An existing example is the stomach cancer gene in various Māori whānau in Tairawhiti and Bay of Plenty, where those carrying the gene are offered surgery to remove the risk of stomach cancer. Interventions are specific to various conditions.

Whānau dynamics

Much like whakapapa, genetics is inherited through generations from our ancestors. Unlike whakapapa, genes are mixed differently between individuals from the parents. This means that an individual can have a very different genetic makeup from their sibling. This is very similar to ira tangata, the essence of the individual.

Despite these vital individual characteristics, whakapapa remains constant, much like ira atua and ira tipuna, while individual DNA has very dynamic elements. So, we are increasingly using the term 'Te Mataira' (the faces of our essence) to describe the richness of genomes, in Māori terms.

Our genes also contribute to who we are and tell us about our connections to each other, and so our DNA sequences have so much to tell us.

Cultural benefit to understanding DNA alongside whakapapa:

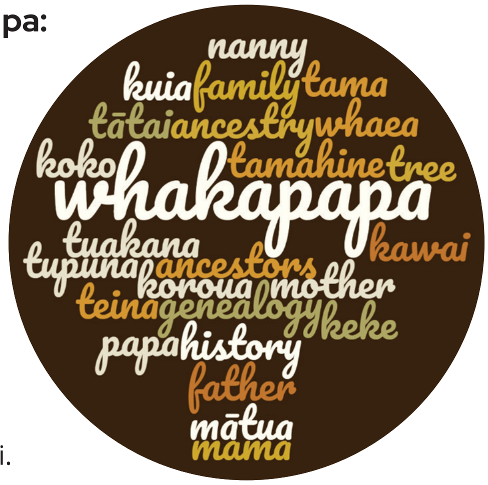

It is important that, much like our whakapapa records, new generations will participate in the care of our biological whakapapa.

A key element of maintaining mana kaitiaki over the data is by creating connection with its origin. Its human, tangata whenua origin grounds the sample and data within its cultural context. By maintaining its point of origin beside the genomic data, we retain and maintain its mana. We also create the pathway for ongoing whakawhanaungatanga (relationship) between the genomic data, the originator and researchers and their institutions. This is the primary practice for long term mana kaitiaki being maintained. This study is also considering what can be done to ensure inter-generational mana kaitiaki.

- The diagrams below use colour to show how genes can have mixed distribution through a whānau. The colours appear random. Each individual is quite different at the genetic level. Most of the human genome is similar across humanity, but this mix of DNA ensures uniqueness and also provides unique points of resilience and vulnerability.

Whānau Dynamics

Whakapapa remains consistent *(ira atua/ira tipuna)* but DNA is dynamic *(ira tangata)*

DNA mixing creates individual uniqueness between siblings

- Is my DNA also your DNA?

- How much is shared?

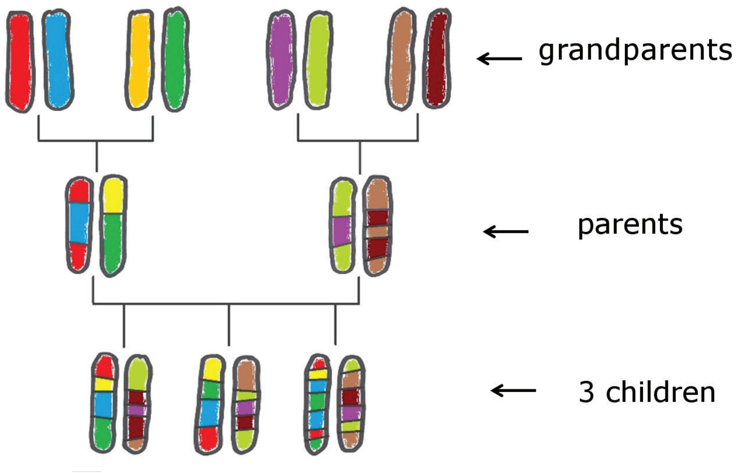

- This last diagram shows how new unions bring both new whakapapa / genealogy as well as new genetic material into the bloodline of a whānau through a broader whakapapa lens. In turn this provides opportunity for new genetic points of resilience and vulnerability.

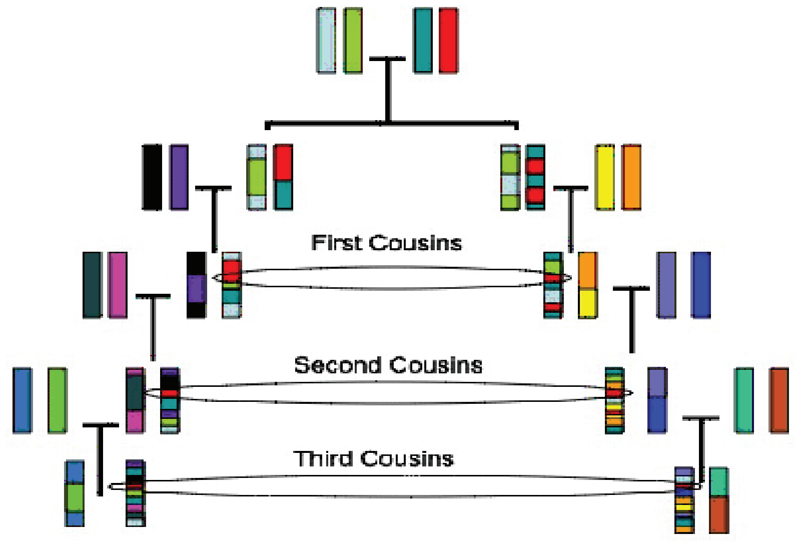

Why does it matter?

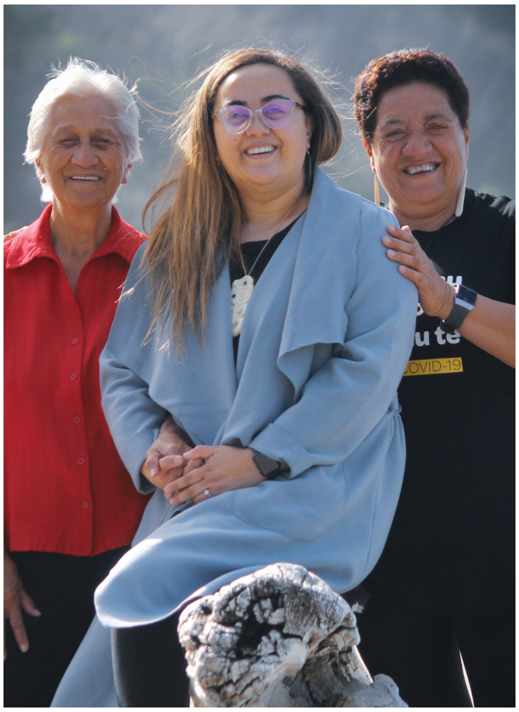

Knowing more about inheritance of genes means we can better understand exactly what each of those genes does. Genomes can help to identify the inherited genetic character of our health – what conditions we have and even what might happen in the future.

For instance, there are now tests that identify changes in your genetic code that help confirm or rule out a suspected disease. These tests can help identify the cause, or they help to work out a person's chance of developing or passing on a genetic disorder to our children. They can also help to find the best treatment options for a disease and to see if some disease risk can be prevented. Overall, genetic medicine is a preventative health opportunity.

Health researchers are studying DNA to begin to answer some of these questions. Some of these tests have helped to identify rare diseases in Māori whānau, or to help doctors prescribe the medicine that will work best for their condition. A lot of this work is being done overseas in non-Māori. What we really want to know is how to best use genetic health tests, for us as New Zealanders.

Ngāti Porou Hauora is seen widely as the lead indigenous agency for indigenous genomics. It has developed a partnered approach to genomic science. Being Māori, building a self-determining, co-design approach is a huge opportunity. We would like to know more about genes in general and their effect on common diseases for us, like heart disease and diabetes for instance. We would also like to understand and introduce best practice approaches towards indigenous people managing their community medicine.

Genetic tests are more accurate and useful if we design them for our circumstances and our people. To do that well, for the benefit of all, we need good information and good processes within Māori community and mainstream institutions. All of this is about improving health.

What we mean by health genetics research
– some examples based on clinical experiences

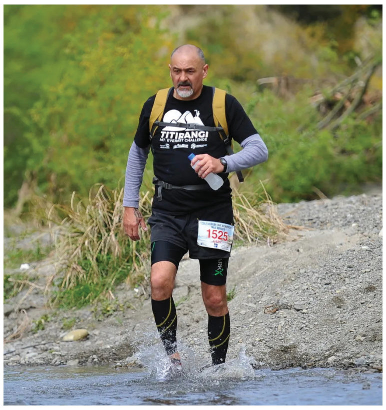

Hemi is aged 55. He has been feeling low and moody for some months now. Recently his whānau have been encouraging him to speak to his doctor about these issues. The doctor listened and told Hemi that she wonders if he might have depression. There are some medicines which can help treat this sort of problem. Hemi is reluctant since he has heard that some of these medicines can have side effects and a friend of his tried similar treatment and the drug his doctors prescribed did not work. However, Hemi had a genetic test to see if it would inform him and his doctor what drugs might have the best chance of working with the least risk of side effects. Four months later Hemi is feeling much better. The drug first prescribed to him worked well and he was not troubled with any major side effects.

When Hine was 22, she suddenly developed seizures. The doctors examined her carefully and carried out tests to check what was going on. After all those tests came back clear Hine was hoping that the seizures would not recur and that she would not have to go on medicines to control them. Unfortunately, she had more fits and her doctor recommended that she start a medicine. Hine knew the drug worked well for her auntie but looked into it and found out that some people who start on this medicine develop a reaction to it. Her doctor knew that too but also knew that a genetic test can be done to predict if that was going to happen to her. Fortunately, the genetic test showed that Hine was a low risk of this reaction, and she was reassured and started taking the drug. Happily, it has been two years now since she last had a seizure, and she can now live a normal life again including driving.

The Rakeiora project

The research is a pilot project. We would like to design a system to combine genetic information with healthcare information, the aim being to make those systems available to other researchers to investigate ways to improve healthcare in Aotearoa. This incorporates co-design with Māori so that any outcomes provide for Te Ao Māori.

Why is that important?

Māori partnership and decision-making success is crucial in developing a New Zealand system that can be trusted. Trust in the next generation health system is equally crucial for all of New Zealand.

> "Māori participation and ultimately a self-determined future has been the promise of the Crown's Treaty guarantees. We are keenly aware that trust must be earned."

We know that disease can be inherited, and we know that information from people's genetic codes is helping researchers better understand disease in humans. New and better medical treatments are being developed through this science. We know that making the best use of genetic information on a person when it is also combined with a patient's medication information. And we know processes that merge both lots of information together will end up being an essential building block for understanding disease, and for improving New Zealand's health services into the 22nd century. We must design systems that better understand and provide for indigenous people's aspirations and genetic character.

What we want to know

We want to work out how to merge whakapapa, genetic and health care information, in a way that is safe, secure, culturally acceptable and respects data sovereignty. And we want to turn that process into a system – basically a way that researchers can use to easily combine completely different lots of separate health and genetic information.

We want to know how this can be done with cultural integrity, facilitating the maintenance of mana tangata, mana tipuna and mana whenua, and with local decision-making for the genomic and whakapapa information.

We call this a pathfinder project – we are trying to explore potential paths to make it easier for other health researchers to follow. While that sounds sensible, it hasn't been done in New Zealand before, and there are a lot of steps needed to bring it all together. To see if that the system works, we need to test it on a small scale first.

So, we want to look at one small part of someone's genetic code simply to demonstrate the potential of a system – that is not to answer a research question about their genome.

How do we go about this?

- We ask a group of people to volunteer to provide their whakapapa and hupe (saliva), and we get DNA.
- We look at a particular gene (CYP2C19) within their DNA.
- We then take that part of the genetic piece of information and look at how we can best combine this with their personal health records securely.
- From that we create a secure place for the data to be stored and accessed.
- Then we work out the best way to report the combined information back to the health professional.
- And we work out how patients access the information and how it should be managed in the future.

What gene are we examining?

We are using the search for one gene as an example of how we go about bringing lots of different information together. There are many thousands of genes we could look at, but we just want to single out one to test the system. This gene has an effect on how well a number of drugs (including a drug commonly used to treat coronary artery disease) work in Māori people.

Individuals who volunteer for our project and have a DNA test will get a report about the gene and how it could affect their response to this heart disease drug treatment. The information (called CYP2C19) will be embedded into their medical records, for future reference.

How will this help researchers?

Precision medicine is a very new way of managing health, one that New Zealand is just starting to explore. To do that, our people need new tools and practices. A process that combines data will give medical researchers such a tool. Understanding and facilitating Māori aspirations and practices will build new behaviours, protocols and processes. It will mean a much better way of using all the information available (including whakapapa) to study other genes that affect health. These methods can be used in different research projects, including ones based on information more specific to our populations, particularly Māori.

> "As a doctor, I want to be as precise as I can be – I want as much information as I can get to get a good picture of what's going on in my patient. I see precision medicine as helping me to do that – it's why I am keen to be at the beginning of this new genomic journey"
>
> **Professor Stephen Robertson**

This is just the beginning. But from that health services can eventually offer more testing options to prevent, diagnose or find the cause of a disease and treat their patients.

Why take part?

Your whakapapa and genomic data belong to you, and you have control of your records through Ngāti Porou Hauora. You will be doing it for your mokopuna sake - understanding options for inter-generational storage and authority relating to their ancestor's genomic information.

A path forward for New Zealand health

Projects like this shows us the potential. Rakeiora is a small study, but one which will ripple out into bigger ones that help us to develop more targeted genetic medicine. Eventually, you might know if you are likely to get a disease, or you might be able to get treatment or preventative options. And you will find out the risk of passing a disease to your children. The Rakeiora project will lay the foundation for this to be a routine part of healthcare in the future - and you will be a part of this journey. NPH and Ngāti Porou are well positioned as leaders and navigators on this journey forward.

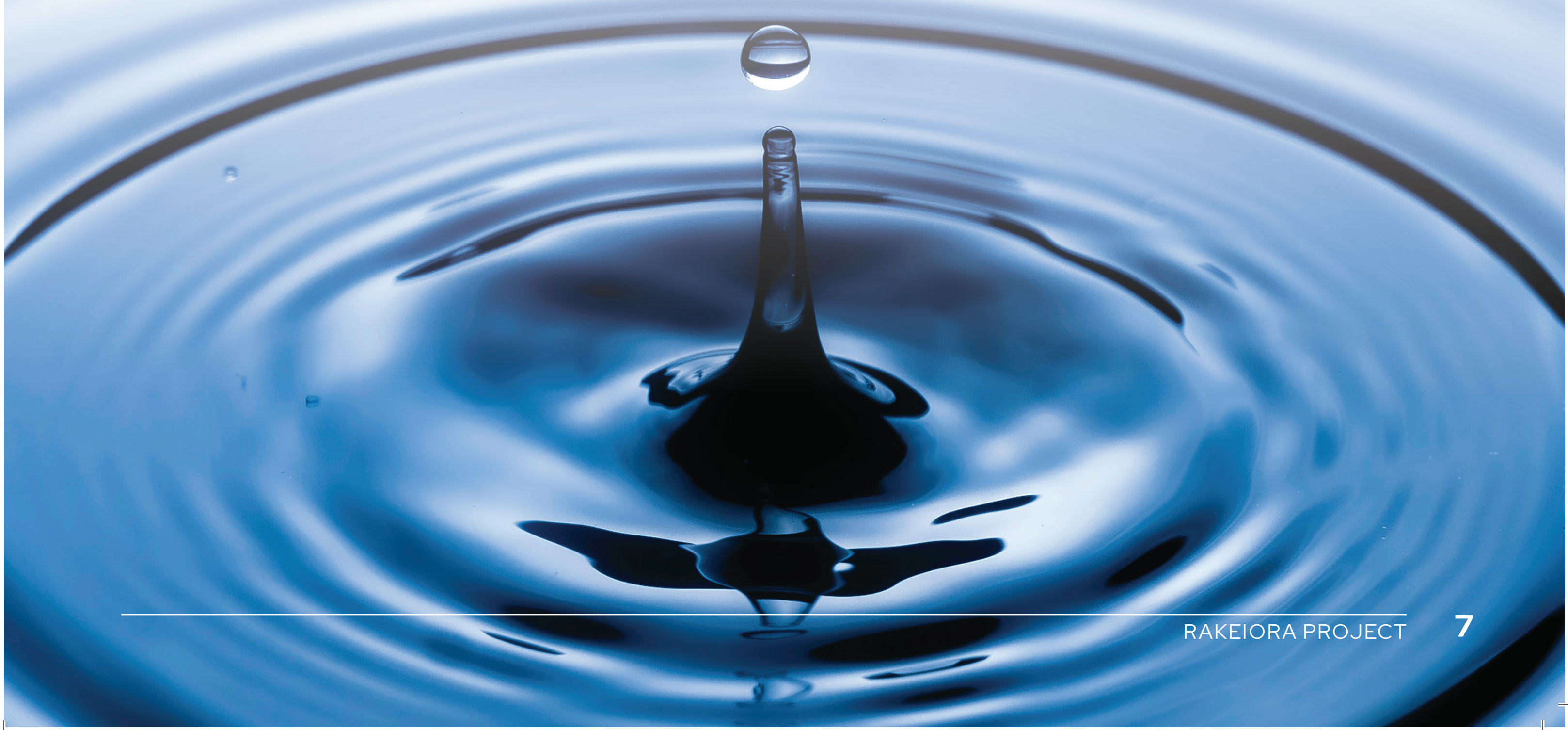

Safety and risks

This is a voluntary study. Whether or not you take part is your choice. If you don't want to take part, you don't have to give a reason, and it won't affect the care you receive. If you do want to take part now, but change your mind later, you can pull out of the study at any time.

Physical risks

For most participants, samples of saliva will be adequate to obtain DNA. There is little risk to the patient from this procedure.

Safety for DNA and saliva samples

There are strict protocols for storing and handling samples, agreed to by Ngāti Porou Hauora and the University of Otago. DNA extracted from samples are stored securely under these provisions. Specimen samples and DNA are treated with the respect, recognising their tapu nature.

Safety for data

Protocols for the electronic storage of data (including whakapapa) are part of the agreement between Ngāti Porou Hauora and the University of Otago. Your data will not be accessed without permission, as outlined in these protocols. Information will not be passed on or sold to other researchers, medical organisations, or businesses, nor commercial organisations. There are safeguards in place to minimise any risk of data loss. DNA samples and data are owned by the participants and that NPH can request their transfer for storage to another secure location should they choose that.

Medical risks

The study is not a genetic test designed to search for the potential for inherited diseases in individual participants. We are simply looking the genome and using it to design and test a system for merging with individual health data and whakapapa. There is therefore very little chance therefore of finding out other health information through this test. CYP2C19 information will be embedded into your medical records.

The genomic data extraction process

Consent given

↓

Saliva sample taken

↓

DNA extracted from sample and stored securely

↓

DNA scanned and digital information produced

↓

Genomic data securely stored

↓

Variants in CYP2C19 gene extracted from Personal Genomes and analysed

↓

Genomic information combined with participant's health information records

↓

Results externally checked for accuracy and validated

↓

Genetic test results on gene embedded into participant's clinical health records and reported to their health professionals

↓

All de-identified results reported to Ngāti Porou Hauora

↓

Guidelines on protocols for personalised medicine research prepared for health research community

FAQ's

What exactly is a pathfinder project?
The proposed research is a pilot project to test a system. We would like to look at one small part of someone's genetic code simply to demonstrate proof-of-principle, not to answer a research question about their genome.

Why is this different from all the other genetic studies Ngāti Porou Hauora is involved in?
You may have been involved and given samples for other research projects. These studies are independent of one another and so have a different approach. But what they all have in common is the aim of improving the accuracy and precision of medical care.

Is there genetic testing in New Zealand?
There is a very small amount of genetic testing being done in New Zealand, but more systems and information are needed to bring some of the techniques being developed overseas.
Genomics Aotearoa plans to build capacity and capability in this area.

What happens to the DNA?
DNA will be extracted from saliva samples provided from participants and used to create a personal genetic code (a genome). Any residual DNA will be stored securely.

Why can't you give us more information, why aren't you looking at all of our genome?
This is only a small pilot study which doesn't provide for broad investigation. The single study is to demonstrate proof-of-principle of this capability, not to answer a research question as such. Because we are only focused on two genes, the chances of finding other clinically important information are extremely low.

Can a participant actually use the data collected on them?
Individual information on these two genes will be on the participant's medical records, held by their individual health provider. The health provider will advise on the results. This is not part of a study into your full genome.

Can the data only be used for this project?
Yes – the consent process makes it clear the data is only for this project.

Can any other organisations or government agencies access the data?
No.

How will taking part in this project benefit me or my whānau personally?
Knowledge on the two genes will help participants and their health provider to consider health management options. In the long-term, we want to develop methods to predict heritability and resistance in genetic conditions and to plan appropriate medical care. This may help to understand more about if a genetic condition is passed on to your children. This project is just the start of that. Importantly, it will help to see the links between genes and health.

Is the data easily useable in a hospital or with my doctor?
No, it is too early to expect the health system to use genomic data like that – it is very specialist information. One of the aims of this project is to make such data useable in the healthcare setting in the future. Much more training is needed across the health system.

Will whoever takes the sample have to sign a legally binding document that ensures that my data cannot be used for any other reason that the research?
Yes.

How long is the study?
The study will take around 18 months. The information we get from you in this project is only being used to test the design of a process to merge genetic and medical datasets and will not be retained when the project finishes.

Will I get paid to participate?
No, but a koha will be offered to recognise the value of your contribution.

Can more than one member of the whānau participate or is it better to have whole whānau contribute?
More than one member of a single whānau can contribute to this project but it is not necessary to have an entire whānau agree to participate.

Why don't you need to take DNA samples from the entire Māori population?
It would be great to do the project on that scale, but we simply don't have enough money!

Introducing our people

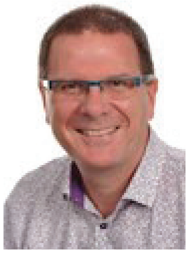

Professor Stephen Robertson is the Curekids Professor of Paediatric Genetics. Educated at the University of Otago, he graduated in Medicine in 1990. He specialised in Paediatrics and Clinical Genetics after training in Auckland, Melbourne and Oxford. His research interests include genetic conditions that affect the development of children and ensuring equity in the delivery of clinical genomics across Aotearoa.

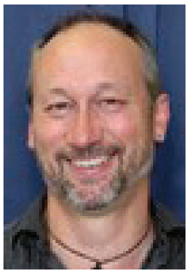

Dr Phil Wilcox, Ngāti Rakaipaaka, Ngāti Kahungunu ki te Wairoa, and Rongomaiwahine) is a Senior Lecturer in the University of Otago's Department of Mathematics and Statistics, with experience in applied genomics and statistics, and works extensively in Māori-related research including development of tikanga-based frameworks for genetics research. He co-leads two genomics-based projects focussing on Māori health, and was formerly a mandated spokesperson for Ngāti Rakaipaaka regarding the Rakaipaaka Health and Ancestry Study. He has worked on genetics of plant species (particularly forest trees) and human diseases. He teaches tikanga (Māori bioethics)-based frameworks in science courses at both graduate and undergraduate levels, as well as statistics and quantitative genetics. He also co-teaches the Summer Internship of indigenous peoples in Genomics (SING) Aotearoa, and is a member of the Health Research Council of New Zealand's Ethics Committee which oversees institutional and regional ethics committees.

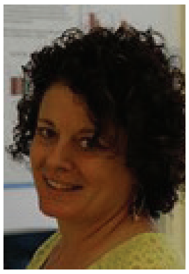

Dr Donia Macartney-Coxson leads the Human Genomics Team at ESR (The Institute of Environmental Health and Research). She fell in love with genetics at High School and has continued that journey (with a few detours along the way) ever since. She is originally from the UK where she did her training and held a couple of research positions at Birmingham University (one looking at lung cancer, another researching obesity) before coming to New Zealand to study gastric bypass and to try to understand why it is so good at treating type-two diabetes. She met her husband, a first generation New Zealander, in Wellington and, as they say, the rest is history. Her research focuses on metabolic diseases (especially obesity and type-two diabetes) and how DNA and our environment (for instance diet, stress) interact in disease through molecular mechanisms called epigenetics. The team at ESR are also passionate about empowering their collaborators/partners to access and explore data in a way which is meaningful for them.

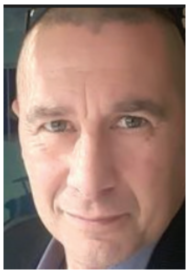

Benjamin Iwikau Te Aika, Bachelor Māori Studies, Master Professional Practice (Economics)

Ko Kati Raureka, Kati Urihia, Kati Puneke, Kati Kaweriri, Kai Te Rakiamoa, (no Kati Wairaki tipuna), Ko Kati Matamata, Kai Tutekawa (no Kati Mamoe, Waitaha hoki), Ko Kati Rakai (no Waitaha) oku take tipuna ki te Wahi Pounamu. Ko Ngāti Mutunga, Ngāti Tama, Te Atiawa oku take tipuna ki te whenua o Taranaki Mauka Ariki. Tihei mauri ora!

Ben has grown up in a family environment strong in whakapapa (genealogical) and whenua Māori (land) traditions. Ben is a former professional soldier, former professional hunter and an accomplished professional Māori carver, these are a strong base for Māori life in the 21st century. He is a former professional environmental advocate, consultant and policy analyst working across multi-disciplined development and environmental projects. This has led to a natural progression to applied Māori economic development a field of passion for the last 12 years. Understanding holistic human development in relation to mana motuhake is his lens in these areas.

Ben has most recently been engaged as a Matauraka Māori specialist at Genomics Aotearoa based at the University of Otago and is now a Programme Manager for a genomic science research programme Rakeiora, funded by the Ministry of Business Innovation and Employment.

Ben lives with his two children Te Waipunahau and Iwikau in Dunedin.

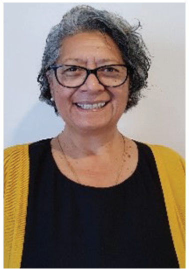

Caroline Koia is the Rakeiora Research Assistant. Ngāti Porou, is a former Lecturer in the Eastern Institute of Technology, Nursing School, Tairawhiti campus, Gisborne. She is a registered nurse and has experience in specialised psychiatric emergency assessment and treatment care after training in Sydney, Australia. Caroline has a Masters of Nursing (Clinical) and graduated at the Victoria University Wellington in 2009.

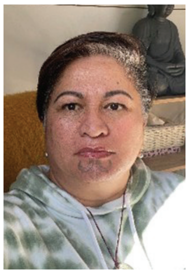

Rhonda Tibble comes to the Rakeiora project as a Ngāti Porou tuturu - home grown product here in the Tairawhiti. Her whakapapa stretches the entire eastern seaboard of the East Coast. 30 years later Rhonda has returned to work at Eastern Institute of Technology in the Te Whatukura Department teaching Te Reo Māori, Tikanga Māori, Māori Models of practice and Treaty of Waitangi discourse. Rhonda also works as a Cultural Facilitator for various projects with Te Ara Poutama o Aotearoa in the Mana Wahine, Womens Strategy, Special Treatment Unit of the Māori Pathways division towards transformative agency of service users and custodial staff alongside Human Centred Design. Rhonda's specialisations are embedding Mātauranga Māori, Mātauranga a Iwi, intuitive wairua practice in to wananga tangata. Helping other to realise their powerful learning potential is always her focus. Rhonda is a current board member of Ngāti Porou Hauora. She sees the relationship of genetics as an important relevant discussion paired with whakapapa critical analysis and medical records as instruments to help Ngāti Porou develop more conscious conversations at the whānau level about our own personal well-being journeys through life. The more we participate in our health realities through robust data, the more informed our choices become. We have to get precise in understanding our well-being and non – well-being factors.

"Whakakahāngia toū oranga."

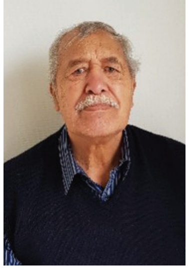

Tuta Niho Niho Haerereoa is a Pou-Tuara / Cultural Advisor for the Te Whare Hauora o Ngāti Porou. His whakapapa connects him to te Whānau a Te Aowera me te whānau a Pokai. Tuta has comprehensive knowledge of the regions local history, growing up during the horse and cart era. Like many young men of his generation, Tuta ventured abroad to explore what the bigger world outside of the east coast had to offer. Throughout his excursions around the motu, he became aware of the state of Māori health. He believes the Rakeiora Project is a stepping-stone to explore how best we can foster a healthier and prolonged life for our people to live longer in this world. "Kia Mataara"

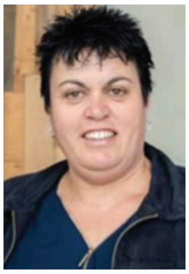

Frances King is the Research Manager for Ngāti Porou Hauora. Ngāti Porou, whānau a Rua ki Tokomaru Bay. Frances comes from a nursing background working locally. She is part of the Leadership group for the Rakeiora Programme. She has also worked as a Professional Fellow for Otago University Hauora Māori, as a Clinical Advisor for Clinical Advisory Services Aotearoa (CASA) and Rural Facilitator for the Rural Health Alliance Aotearoa New Zealand (RHAANZ). She is a strong advocate for rural health services.

Contact

Caroline Koia, NPH Research Assistant

Te Rangawairua o Paratene Ngata Research Centre,
Ngāti Porou Hauora

Email: caroline.koia@nph.org.nz
Phone: 021 392 772

Frances King, NPH Research Manager

Ngāti Porou Hauora

Email: Frances.king@nph.org.nz
Phone: 021861426

genomics aotearoa

University of Otago, Te Whare Wānanga o Otāgo, New Zealand

The University of Auckland, Te Whare Wānanga o Tāmaki Makaurau, New Zealand

E/S/R Science for Communities

Nō whea hoki te ingoa o Rakeiora?

I tonoa e Ngāti Porou Hauora māna e tapaina he ingoa mo te hotaka rangahau a Ira tangata nei I ahu mai I te tari kawanatanga a MBIE me ona hoa kaipakihi ko ESR, ko Te Whare Wānanga o Otakou, ko Te Whare Wānanga a Tamaki Makaurau.

Ko Rakeiora te ingoa I tohua e Herewini Parata Tiamana o TRONPnui me te āwhina ā rangahau nei e Huti Puketapu - Watson mema Pōari o Hauora Ngāti Porou.

He tipuna rahi tonu a Rakeiora[1] I haramai aia I Hawaiiki nui. Ko tona rongo I puta pakari mai ai I Poronihia ki te rawhiti ahu atu ki Niu Tireni nei.[2] Kei roto anō hoki te tipuna nei a Rakeiora I te nuinga o ngā tatai whakapapa o ngā iwi o te taitamawahine o te ika a Maui. E kīngia ana ko Rakeiora; he matakite, he tohunga taiao hoki.

Toi
Rauru
Tahauri
Tahatiti
Ruatapu
Rakeiora[3]

Ki te wetewete I te ingoa o Rakeiora e marakē ana te kite I te huahua o te tohu o roto. He whakapiringa ki te kaupapa hauora nei te whaitanga atu o te ruku hohonu I te mātauranga pūtau-ā-ira a ngā uri o Ngāti Porou kia rapaina I te hua nui o o rātou Toiora ake hei ngā rangi kei te heke mai.

Ko "Rā": koia te whitinga mai o Tamanui te rā o te ia rā o te tangata. **Ko "Kei":** te wāhanga ki te ināianei, I te wā e tū nei te tangata ki te hora. **Ko "Ora":** koina kē te pūkahu o te mauri ora I whoatungia e Tane ki a Hineahuone kia ora mai te ira tangata ki te whaiao, ki te ao turoa o te taiao e noho nei tātou hei tangata.

Tērā te wāhi ki a Ruatapu[4] te hoariri tungane a Paikea. He nui ngā kōrero heke iho[5] ki ngā uri o Ngāti Porou ahakoa te weriweri, ahakoa te taumaha ka āhei te puta I te kino ki te pai engari rautakitia te puta.

Nā rā, koia kē te hohonutanga hei whakapiringa ki te kaupapa mātauranga pūtaiao o te pūtau-ā-ira a Rākeiora e whakapapatia nei ki a Ngāti Porou.

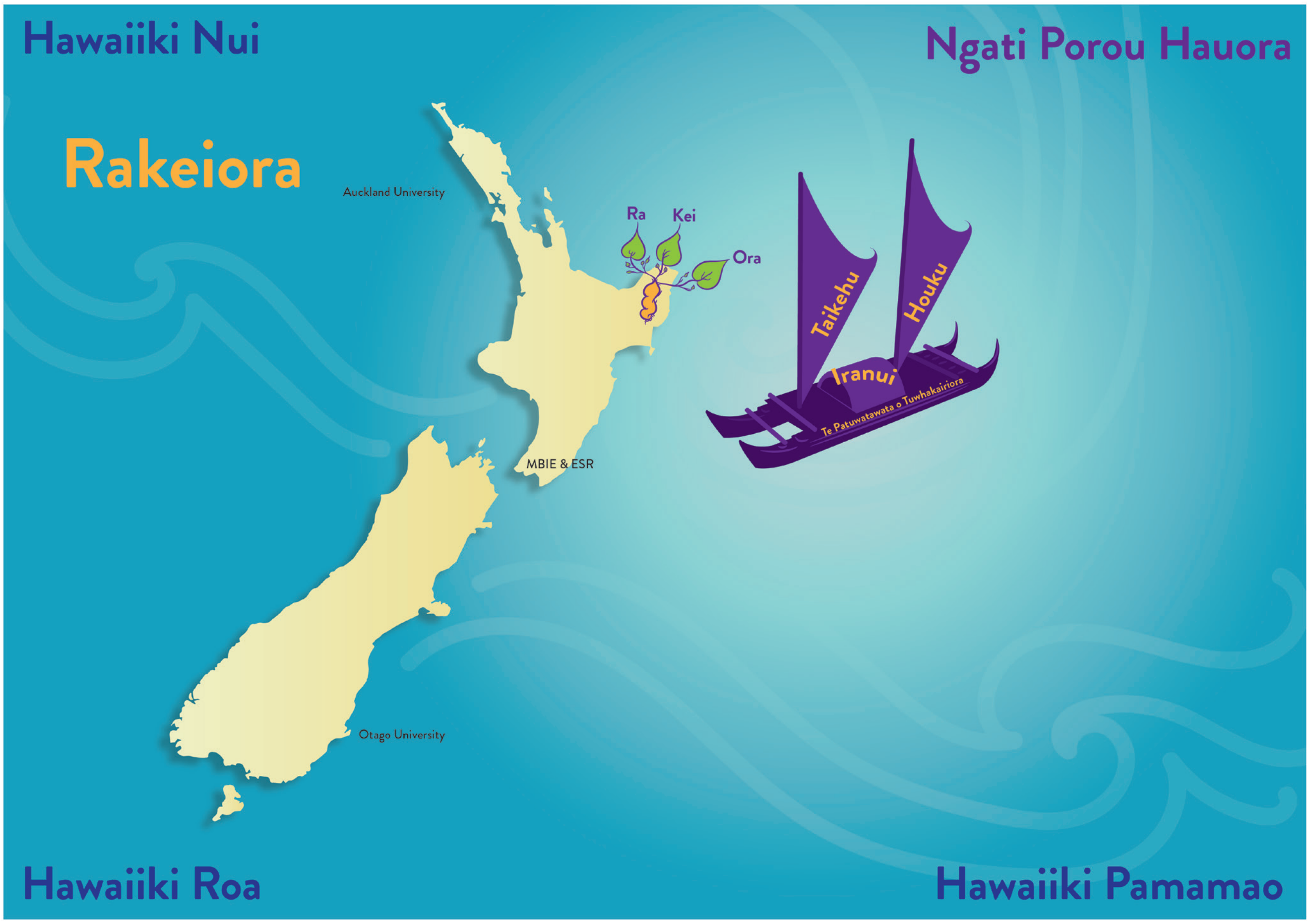

CREDIT: **Melanie Tahata** (Ngati Uepohatu, Ngati Porou, Te Aitanga-a-Hauiti me Ngai Tamanuhiri)

1 In Line 27 of the Oriori Po! Po! https://paperspast.natlib.govt.nz/periodicals/TAH196512.2.12 pp.19

2 Taonui,R. (2011) in Te Ara Encyclopedia.

3 Ngata, A.T (1977). *Rauru-Nui-A-Toi Lectures:* Lecture 6, pp.1. Informant is Wi Tahata

4 Reedy, A. (Trans. & Ed.). (1993). Nga korero a Mohi Ruatapu: Tohunga rongonui o Ngati Porou: The writings of Mohi Ruatapu. Canterbury University Press. https://doi.org/1 O. l 8124/ed3c-jm64 pp.142

5 Noho Noa Whatitata Ngā Mōteatea,II,pp64-67.

He aha I whiria ko te Waka Hourua hei āria whakapupuri I te mana o Rakeiora e ai ki tā te tirohanga hauora o Ngāti Porou?

Nā te waka hourua a tātou tipuna I haere mai ki Aotearoa nei. Ko te hekenga nui tēnei mai Hawaiiki nui, Hawaiiki roa, Hawaiiki Pāmamao mā runga te moana nui a Kiwa.

Ko Nukutere, ko Horouta, ko Takitimu, ko Te Ikaroa a Rauru, ko Tereānini, ko Mataatua ngā waka o ngā uri o Ngāti Porou. Hei tā Te Rangihīroa ko te iwi Māori ngā Paekīngi o Te Aranga mai o Te Rā[6].

He nui tonu ngā āria hauora Māori[7] kua whakawhenuahia e te waka hourua hei tūāpapa whakapiringa I te whakaaro māori ki ngā kaupapa here I ngā whakarātonga kāwanatanga.

I kōnei mō Rakeiora ki Ngāti Porou ka whakahangai atu te waka hourua kia huaina ko te **Kakano korau a Iranui**[8] a ko tōna mana taua koia ko te **Pātūwatawata**[9] **o Tūwhakairiora.**

Ma runga I a Iranui rāua ko Tuwhakairiora I hua mai ai ngā whakapapa whānui o Ngāti Porou. Mai I a Rakeiora I ngā kawai heke o Toi Te Huarahi tae atu ki a Iranui rāua ko Tuwhakairiora ngā mokopuna heke iho I a Porourangi. Kāti hā koinei ngā hekenga kawai o Ngāti Porou e tū nei te rangi nei.

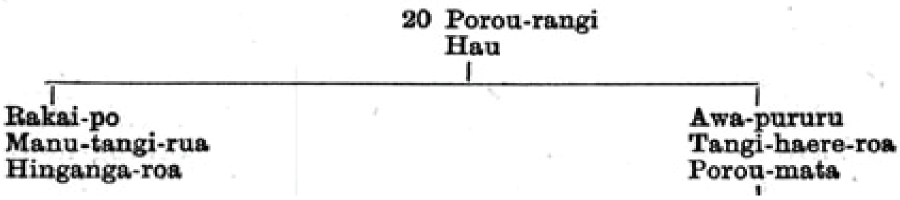

[10]

Hingangaroa = *Iranui* **Materoa** **Tawhipare** **Te Ataakura**
Taua Mahaki Hauiti ***Tuwhakairiora***

Tā te wahine he tiaki I ngā uri, he whakatipu, he ukaipō. Kei a ia te mana whenua, me te mana tangata. Tā te tane he tiaki tona wahine, tona whenua. Arā te kōrero, he wahine he whenua ka ngaro te tangata. Ko **Iranui** te **whare whakaahuru o te Pūtau**-ā-Ira o Ngāti Porou Hauora, ko **tona toa tiaki** ko **Tuwhakairiora** me tona pā tuwatawata.

Ko te āria o te pā tūwatawata he taiapa here I te kakano korau a Iranui. Koira ngā ira o te hunga ka tangohia o rātou ira kia tataringia e UoA rāua ko UoO I roto I te hotaka rangahau hauora nei a Rakeiora.

Ko ona ama ko **Te Tikanga Ngāti Porou** me te **Te Tikanga Pākehā**. Koianei te hononga atu ki a MBIE, ESR, University of Otago, University of Auckland ki a Ngāti Porou Hauora roto I tenei hotaka rangahau hauora.

Ko ona tēra ko **Te Tipi o Taikehu** rāua ko te **Tone o Houku** I tapaina mō ngā tihi tapu e rua, kotahi te mea tane, kotahi te mea wahine. He tūtohu whenua ka whakaatūria I te mana rite tahi o te tane me te wahine e ai te tirohanga Ngāti Porou o nehe, o naianei hoki.

He aha te hononga o ēnei ingoa Ngāti Porou ki te hotaka hauora pūtau-ā-ira o Rakeiora?

Tuatahi: mā raro I te ingoa o Rakeiroa ka tataitia atu ki te whakapapa o tēnei tipuna nui o Hawaiiki hei whakapiringa heke iho ki a Ngāti Porou me ana uri o naianei.

Tuarua: Ko ngā whakaingoatanga o te whakahoahoa o Rakeiora ki Ngāti Pōrou tētahi peka o ngā mahi rangahau hauora kua uru atu e Ngāti Porou Hauora hei rapa I te whano riakina o te rongoa tangata. Mā reira he huarahi e anga whakamua atu mo ngā momo mate ka pangia ki te pūtau-ā-ira o te tangata Ngāti Porou. He kimi I te tapu, I te tipua, I te toiora te mahi nei.

Tuatoru: Mā te whakakōpani ake I ngā āria whakaaro nei ki Te Ao Mātauranga Ngāti Porou ka taiapa herengia ki te whenua, ki te tangata. Kāorehā ki te pūkahu o te mana motuhake o Ngāti Porou I ōna whakaterenga kaupapa rangahau kia eke ai ōna uri ki te Toi o ngā rangi.

6 Buck, P.H (1954). *Vikings of the sunrise*. Whitcombe and Tombs. Christchurch.

7 Elder,(2013);Rata, Hutchins & Liu,(2012); Pitama, Huria, Lacey,(2014).

8 Williams, J. (2010) in Mahika Kai JPS 119-2-04. Best (1976: 273) mentions the Ngāti Kahungunu saying: “Te kakano korau a Iranui”, which attributes the korau to Iranui (sister of Kahungunu) who introduced the seeds to Uawa (Tolaga Bay) over 20 generations ago. As Best pointed out, such a small seed would normally be referred to as purapura, rather than kakano. Ngāti Porou also refer to korau being available in traditional times (Best 1976: 274). Ngāti Kahungunu also have other stories that tell of korau growing around precontact settlements

9 Fortified palisade.

10 http://www.jps.auckland.ac.nz/document//Volume_3_1894/Volume_3%2C_No.4%2C_December_1894/The_Maori_tribes_of_the_East_Coast_of_New_Zealand%2C_by_W._E._Gudgeon%2C_p_208-219/p1 ref pp218

Hei reira te whakatauāki a Tā Apirana Ngata a "E tipu, e rea"[11] e whanake haere ake nei.

Ko **ngā rau kumara e toru** te mana whakahaere o tēnei rautaki hauora a Ngāti Porou me ōna hoa whakawhenua a MBIE, a ESR, a UoO, a UoA:

NGA RAU KUMARA E TORU o RAKEIORA Ki NGĀTI POROU

**Ra**	Whakapapa	Genealogy
**Kei**	Pūtau -ā-ira a te Tangata	Gene Code
**Ora**	Ngā Rikōata Hauora o Te Tangata	NHI

Ko ngā rau kumara e toru nei ka rarangahia hei whakakitenga ka pēwhea te toiora o te tangata. Ka hua mai te whakawhenumitanga o nga rau kumara e toru hei ara whakakite I whano riakina ōhau nā mate hauora hei ara whakakite māhau ki te ora. Rau Tipu, Rau Ora[12], Rau Tangata Rūnanga mai tātou e.

Bibliograhy:

Elder H. Te Waka Oranga: An Indigenous Intervention for Working with Māori Children and Adolescents with Traumatic Brain Injury. Brain Impairment. 2013 November:1-10.

Rata A, Hutchings J, Liu J. The Waka Hourua Research Framework: A Dynamic Approach to Research with Urban Maori Communities. The Australian Community Psychologist. 2012 June;24(1):11.

Williams, J. (2010) in Mahika Kai JPS 119-2-04.

11 https://www.nzcer.org.nz/nzcerpress/early-childhood-folio/articles/e-tipu-e-rea-messages-early-childhood-practice

12 https://trusttairawhiti.nz/assets/Uploads/tairawhiti-recovery-plan-rau-tipu-rau-ora-may-2020-v16.pdf

ENGLISH TRANSLATION

Where did the name Rakeiora come from?

Ngāti Porou Hauora were invited to provide a name for the genome project. Our business partners in this work are MBIE, ESR , UoO and UoA. Our TRONPnui Chairman Herewini Parata provided the name Rakeiora. Huti Puketapu - Watson and Rhonda Tibble provided further kōrero.

Rakeiora was a significant ancestor who came from the great Hawaiiki. He was known throughout east Polynesia and New Zealand. He also features in the eastern seaboard tribes whakapapa of the North Island of New Zealand. The following whakapapa shows his descent from the eponymous ancestor Toi.

Toi
Rauru
Tahauri
Tahatiti
Ruatapu
Rakeiora[1]

When dissecting the name Rakeiora it becomes clear that there are significators inside the name itself. These significators a point to health concepts pertaining to deeper collusions of genetic research for Ngāti Porou descendants in search of greater wellbeing health outcomes for their future personal prosperity.

Ra: refers to the rising of Tamanui te ra, the sun that rises daily. **Kei**: refers to the present tense time locator of now, where one finds themselves in a space, place and time continuity. **Ora**: refers to the abundance of a living, thriving life force, that which Tane (God, Who created humans) put in to Hineahuone so that humanity would prosper in the living material worlds of this human realm.

We cannot dismiss the place of Ruatapu in this whakapapa or his sworn enemy sibling Kahutiaterangi (also known as Paikea). There are many repositories of kōrero about this seminal event that led to being rescued by a Whale and brought to shore in New Zealand. However, what this story reminds us of is the importance of resilience and endurance in difficult times. How to get out of bad situations and in to better ones through strategic intention and action.

This is an important alliance opportunity that the Rakeiora Genomic project brings to Ngāti Porou future precision medicine outcomes.

Why has the Waka Hourua been selected to represent Rakeiora in the Ngāti Porou health model lens?

Waka Hourua were the mode of transport used to get our ancestors to New Zealand. This period was known as the great migration from the big Hawaiiki, the long Hawaiiki, the distant Hawaiiki over the great Pacific Ocean.

Our waka specific to the descendants of Ngāti Porou were: Nukutere, Horouta, Takitimu, Te Ikaroa a Rauru, Tereānini, and Mataatua. According to Sir Peter Buck, we Māori are the Vikings of the Sunrise.

There are a number of Māori Health models that use waka hourua to ground theory as a foundation for Māori paradigms of thought used in the supply of government policy in to practice today.

Here for Rakeiora's partnership to Ngāti Porou we have aligned and attributed the waka hourua names that ground Ngāti Porou specific referenced bodies of Mātauranga a Iwi.

1 Ngata, A.T (1977). *Rauru-Nui-A-Toi Lectures:* Lecture 6, pp.1. Informant is Wi Tahata

Namely the Kakano Korau a Iranui and its associated protectorate body the Pātūwatawata of Tuwhakairiora. Upon Iranui and Tuwhakairiora all the wider whakapapa lines of Ngāti Porou come together. Similarly, those of Rakeiora through the Toi Te Huatahi lines converge the other whakapapa binaries to Iranui and Tuwhakairiora. Rakeiora landed here and Iranui and Tuwhakairiora were born and bred here in New Zealand. Therefore cementing the whole gambit of Ngāti Porou whakapapa origins to present day. See Whakapapa diagram below.

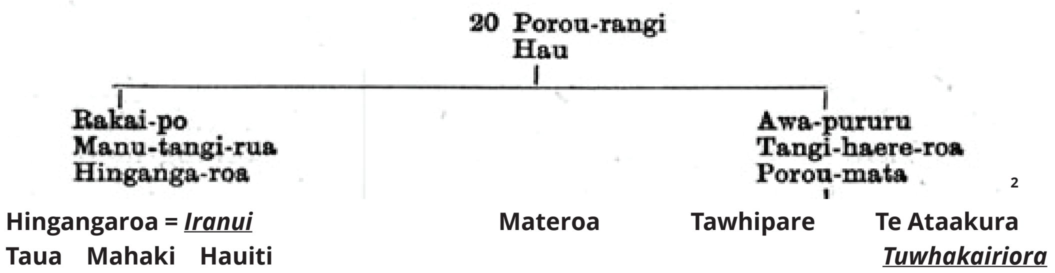

The role of the women is to care for the descendants, to raise them, as their mother. She has the land right and people right. The role of men is to care for and protect their women and land. As quoted generically, "for women and land men must die".

In this model Iranui is the safe repository of genetic material of for Ngāti Porou Hauora, her warrior protector is Tuwhakairiora and his fortified pā.

The fortified pa concept is a force field for the repository of Iranui. This gene material comes from the participants who agree to have their material extracted and analysed as part of the Rakeiora Genome Project.

The skids of the Waka Hourua represent the **Tiriti o Waitangi Partnership** in action. This is the relationship of Ngāti Porou Hauora with its business partners, MBIE, ESR, University of Otago and the University of Auckland.

The sails are **Te Tipi o Taikehu** and the **Tone o Houku** they are named for the two sacred peaks on Mount Hikurangi being masculine and feminine energies. They are landmarks that display the balance of men and women in the ancient and present day Ngāti Porou perspective.

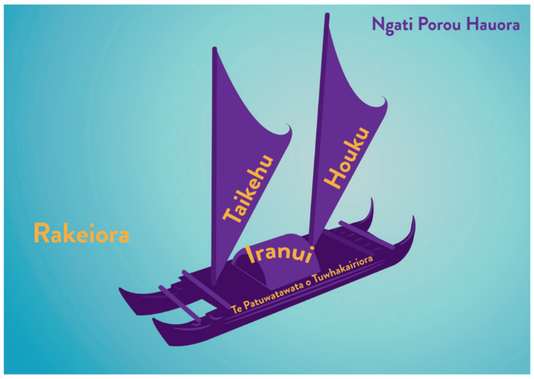

CREDIT: Melanie Tahata

What is the relationship of these Ngāti Porou names to the Rakeiora Genomic project?

Firstly; underneath the banner of the tipuna name Rakeiora we can whakapapa to him as an ancestor who came from Hawaiiki whose descendants' are Ngāti Porou right now.

Secondly; the names in the diagram of Rakeiora Ngāti Porou ground one branch of health research products that Ngāti Porou Hauora is engaged in focusing on better well-being outcomes for its patients.

Thirdly, by containing the concepts in a Ngāti Porou world view the work is understood through ideas that make sense to the people of the land and iwi. Subsequently, connecting to the mana motuhake of Ngāti Porou Hauora and its long enduring research agenda for the providence of its people. This is aptly expressed in Sir Apirana Ngata's aphorism of "E tipu, e rea" of encouraging continual growth and development.

The three kumara leaves are the main operators of this Ngāti Porou health research strategy in partnership with MBIE, ESR, UoO and UoA.

The three kumara leaves of Rakeiora Ngāti Porou.

**Ra**	Whakapapa	Genealogy
**Kei**	Pūtau -ā-ira a te Tangata	Gene Code
**Ora**	Ngā Rikōata Hauora o Te Tangata	NHI

The three kumara leaves weave a remote viewing opportunity to predict how the well being of an individual might pan out. The result of the combination of the three points of reference allow focused attention upon personal well being attributed to your most likely areas of health concern towards precision medicine outcomes. Growth, Well Being, thriving people make for a thriving iwi.

2 http://www.jps.auckland.ac.nz/document//Volume_3_1894/Volume_3%2C_No.4%2C_December_1894/The_Maori_tribes_of_the_East_Coast_of_New_Zealand%2C_by_W._E._Gudgeon%2C_p_208-219/p1 ref pp218

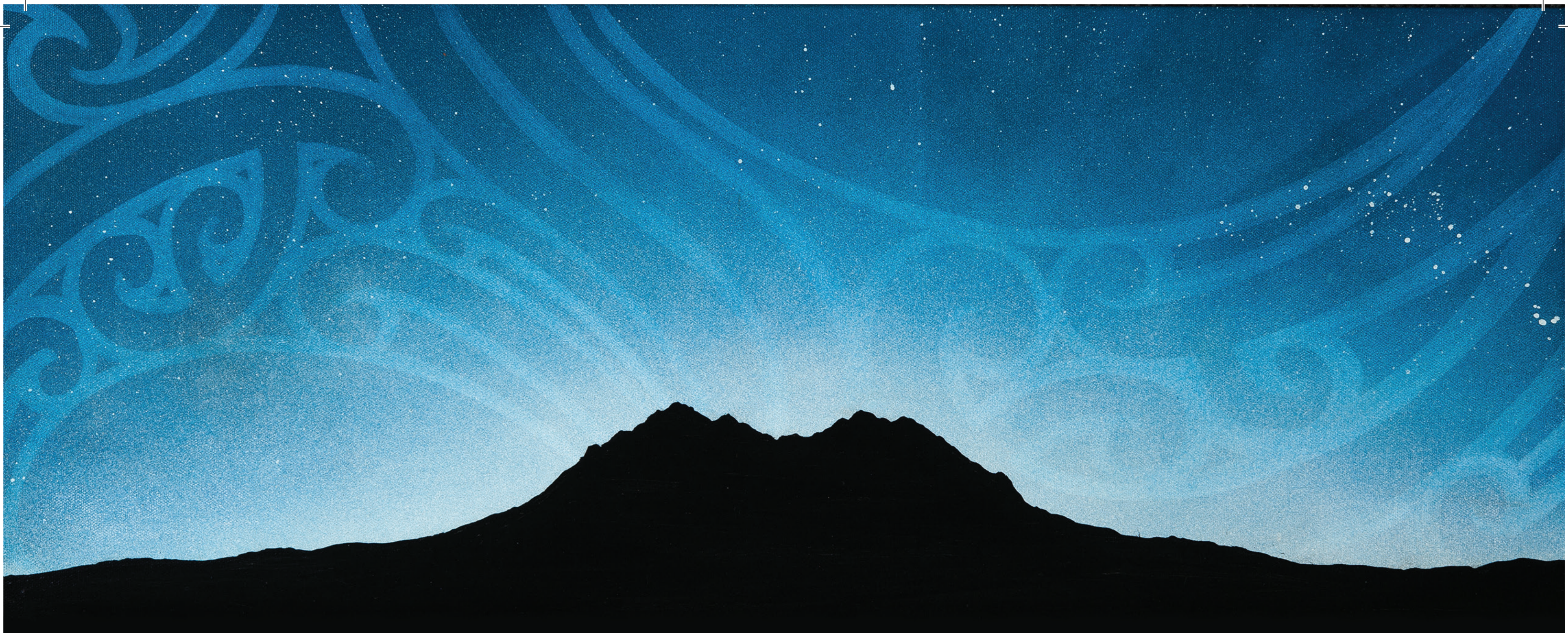

Contact

Caroline Koia, NPH Research Assistant
Te Rangawairua o Paratene Ngata Research Centre, Ngāti Porou Hauora

Email: caroline.koia@nph.org.nz
Phone: 021 392 772

Frances King, NPH Research Manager
Ngāti Porou Hauora

Email: Frances.king@nph.org.nz
Phone: 021861426
