# Supplementary material for: A primary care pharmacogenetic precision medicine pilot based on specific Māori tribal ethical frameworks and principles
Source: J Community Genet. 2026 Jun 28;17(4):79. doi: 10.1007/s12687-026-00914-7 (PMC13310208; doi:10.1007/s12687-026-00914-7)
Supplement: Supplementary file 2 — Supplementary Material 2: File 2. Questionnaire for Participants of the Study [file 12687_2026_914_MOESM2_ESM.docx]

**
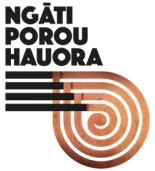
**
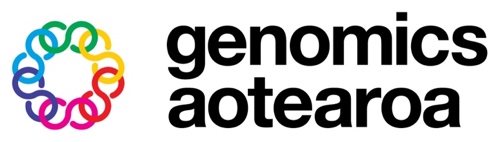


**Rakeiora - A Pathfinder for Research into Genomic Medicine**

**SURVEY**

**Name** (please print clearly): _________________________ **Date of Birth:** **DD / MM / YYYY**

**Data of Survey DD / MM / YYYY**

To safely use databases like this in healthcare research, it will be necessary to establish tikanga to control how, for what and by who the data are used. We would like to have a conversation about your perspectives and opinions on these matters.

None of your replies will commit you in any way to how your data is used in the future. All we wish to do with this survey is begin to understand your viewpoint on these matters.

A key factor we wish to understand is whether you personally expect to grant consent every time a researcher would like to use your data for another study or whether other Māori health and/or genomics experts could make that consent decision on your behalf. These requests could be about research related to a current project you have consented for, or it might be about unrelated clinical research.

1. Would you consider giving consent for your data collected for this study to be used in the same way in other research projects that involve linking your clinical and genetic data to improve healthcare (this is termed "precision medicine") in the future? YES / NO

2. Would you consent to your data collected for this study continuing to be used for research after your death? YES / NO

**If you are open to the possibility of also giving consent for use of your data collected for this study in future research, what protections would you require to be in place, for example:**

3. Would you wish to give personal consent every time a researcher wanted to use your data for future precision medicine research? YES / NO

4. Would you prefer a national Ethics Committee and the Ngāti Porou Hauora Board to consent on your behalf to such requests to use your data, as part of their processes for approving future precision medicine research? YES / NO

5. Would you only consent to such studies if the proposed studies had been approved by an Ethics Committee and the Ngāti Porou Hauora Board, provided that the Ethics Committee and/or the NPH Board had advice from a roopu of Māori who are expert in Māori health research involving genomics? YES / NO

6. Would you be comfortable to share your data and information with researchers overseas for them to use it in precision medicine research? YES / NO

7. Do you have any other thoughts about the characteristics an entity would need for you to be comfortable to ‘authorise’ them to approve on your behalf any future use of your data for other precision medicine studies?

If so what are these?

Thank you for your time and thoughts on this kaupapa.
